# Comparative Proteomic Analysis by iTRAQ Reveals that Plastid Pigment Metabolism Contributes to Leaf Color Changes in Tobacco (*Nicotiana tabacum*) during Curing

**DOI:** 10.3390/ijms21072394

**Published:** 2020-03-31

**Authors:** Shengjiang Wu, Yushuang Guo, Muhammad Faheem Adil, Shafaque Sehar, Bin Cai, Zhangmin Xiang, Yonggao Tu, Degang Zhao, Imran Haider Shamsi

**Affiliations:** 1 State Key Laboratory Breeding Base of Green Pesticide and Agricultural Bioengineering, The Key Laboratory of Plant Resources Conservation and Germplasm Innovation in Mountainous Region (Ministry of Education), Guizhou University, Guiyang 550025, China; wushengjiang1210@163.com; 2 Key Laboratory of Molecular Genetics/Upland Flue-cured Tobacco Quality and Ecology Key Laboratory, Guizhou Academy of Tobacco Science, CNTC, Guiyang 550081, China; yshguo@126.com (Y.G.); bincaiuk@gmail.com (B.C.); xiangzhangmin@126.com (Z.X.); yc12101875@126.com (Y.T.); 3 Department of Agronomy, College of Agriculture and Biotechnology, Zijingang Campus, Zhejiang University, Hangzhou 310058, China; 11516093@zju.edu.cn (M.F.A.); 11816126@zju.edu.cn (S.S.); 4 Guizhou Academy of Agricultural Sciences, Guiyang 550006, China

**Keywords:** *Nicotiana tabacum*, ultrastructure, postharvest physiology, pigment metabolism, iTRAQ

## Abstract

Tobacco (*Nicotiana tabacum*), is a world’s major non-food agricultural crop widely cultivated for its economic value. Among several color change associated biological processes, plastid pigment metabolism is of trivial importance in postharvest plant organs during curing and storage. However, the molecular mechanisms involved in carotenoid and chlorophyll metabolism, as well as color change in tobacco leaves during curing, need further elaboration. Here, proteomic analysis at different curing stages (0 h, 48 h, 72 h) was performed in tobacco cv. Bi’na1 with an aim to investigate the molecular mechanisms of pigment metabolism in tobacco leaves as revealed by the iTRAQ proteomic approach. Our results displayed significant differences in leaf color parameters and ultrastructural fingerprints that indicate an acceleration of chloroplast disintegration and promotion of pigment degradation in tobacco leaves due to curing. In total, 5931 proteins were identified, of which 923 (450 up-regulated, 452 down-regulated, and 21 common) differentially expressed proteins (DEPs) were obtained from tobacco leaves. To elucidate the molecular mechanisms of pigment metabolism and color change, 19 DEPs involved in carotenoid metabolism and 12 DEPs related to chlorophyll metabolism were screened. The results exhibited the complex regulation of DEPs in carotenoid metabolism, a negative regulation in chlorophyll biosynthesis, and a positive regulation in chlorophyll breakdown, which delayed the degradation of xanthophylls and accelerated the breakdown of chlorophylls, promoting the formation of yellow color during curing. Particularly, the up-regulation of the chlorophyllase-1-like isoform X2 was the key protein regulatory mechanism responsible for chlorophyll metabolism and color change. The expression pattern of 8 genes was consistent with the iTRAQ data. These results not only provide new insights into pigment metabolism and color change underlying the postharvest physiological regulatory networks in plants, but also a broader perspective, which prompts us to pay attention to further screen key proteins in tobacco leaves during curing.

## 1. Introduction

Tobacco (*Nicotiana tabacum*) is an extensively investigated model plant and one of the most widely cultivated non-food crops. Given its agricultural importance, tobacco is grown in more than 100 countries for its foliage, mainly consumed as cigarettes, cigars, snus, snuff, etc. The plant organs, including fruit, flowers, and leaves, undergo a series of complex physiological and biochemical changes when they detach from their mother plant [1,2,3]. Curing is the process of transforming raw materials into target requirements through certain processes. Color is one of the quality factors of cash crops and agricultural products, and color change associated with carotenoid and chlorophyll metabolism is one of the most obvious phenomena in postharvest vegetative organs during curing and storage. A substantial amount of research has been carried out to comprehend this fundamental postharvest physiological process to improve the commercial value of agricultural goods during the past few decades [4,5,6].

The carotenoids represent the most widespread group of pigments in nature, with over 750 members and an estimated yield of 100 million tons per year [6,7,8], and many carotenoid compounds have been examined, such as *β*-carotene, lutein, violaxanthin, and neoxanthin [9,10,11]. Certain plant proteins, such as carotenoid cleavage dioxygenases (CCDs) and violaxanthin de-epoxidase (VDE), significantly participate in the regulation of the carotenoid and degradation products content in plants [6,10,12]. Additionally, lipoxygenase (LOX) is an important enzyme that catalyzes the co-oxidation of *β*-carotene and plays a significant part in the deterioration of *β*-carotene levels [13,14], while peroxidase (POD) is involved in the cleavage of various carotenes, such as xanthophylls and apocarotenals, to flavor compounds [15]. Carotenoid degradation products are important volatile flavor components and precursors for plant growth regulators such as the phytohormone abscisic acid (ABA) and strigolactones in a range of plant species [10,12,16].

In addition, chlorophyll metabolism is an important biological phenomenon, and it has been estimated that about one billion tons of chlorophyll are destroyed on a global scale each year [17,18]. Chlorophyll compounds mainly include chlorophyll *a* and chlorophyll *b* in plants [17,18,19]. Proteins, such as chlorophyllide-a oxygenase (CAO) and chlorophyllase (Chlase), are involved in the chlorophyll biosynthetic pathway and the chlorophyll breakdown pathway [17,18,20]. Chlorophyll degradation is a highly controlled sequential process that converts the fluorescent chlorophyll molecules into non-fluorescent chlorophyll catabolites (NCCs), which are stored within the vacuole in a range of plant species [18,21,22]. Furthermore, chlorophyll serves as a precursor for important volatile flavor components such as phytol and neophytadiene [16].

Parameters of the CIEL*a*b color coordinate include lightness L* (positive white and negative black), and two chromatic components a* (positive red and negative green) and b* (positive yellow and negative blue) [2,11]. Color change is one of the most dramatic events occurring in plant postharvest organs during curing and storage [2,5,23]. Pigments are compounds that absorb subsets of the visible spectrum, transmitting and reflecting back only what they do not absorb, and causing the tissue to be perceived as the reflected colors [24]. Carotenoids are pigments that range in color from yellow through orange to red, resulting from their C_40_ polyene backbone [6,14]. The green color changes to orange and red due to the breakdown of chlorophylls and the accumulation of the orange *β*-carotene and the red lycopene in plants [6,25]. The plants seem intensely yellow due to the accumulation of the xanthophylls, namely lutein, neoxanthin, and violaxanthin [11,25,26]. The color change is determined by a dynamic shift in pigment composition and their contents in plants, which is associated with the regulation of differentially expressed proteins (DEPs). Proteins involved in photosynthesis, glyoxylate metabolism, carbon and nitrogen metabolism, anthocyanin biosynthesis, protein processing, and redox homeostasis are crucial for color regulation in plants [8,27,28,29]. Moreover, leaf color change is a complex programmed process that is closely related to pigment metabolism and is regulated by fine-tuned molecular mechanisms [8,27,30].

Tobacco is the most important non-food agricultural economic crop and serves as a model plant organism to study fundamental biological processes [31,32]. In tobacco, leaf senescence during postharvest processing is different from natural senescence, and rather an accelerated one [16,23]. It is worth emphasizing that energy metabolism, photosynthesis, jasmonic acid biosynthesis, cell rescue, and reactive oxygen species scavenging are crucial for leaf senescence, and induced leaf senescence may be involved in nutrient remobilization and the cell viability maintenance [33,34,35,36]. Strikingly, carotenoid, and chlorophyll metabolism associated with color change was one of the most important biological processes in tobacco leaves during curing and senescence [23,36,37]. Fresh tobacco leaves are harvested and processed into flue-cured tobacco raw material in a bulk barn. This curing process of tobacco leaves can be divided into the yellowing stage, the leaf-drying stage, and the stem-drying stage. The yellowing stage is the first key step associated with carotenoid and chlorophyll metabolic and color changes in tobacco leaves [16,23]. Thus, studying plastid pigment metabolic and color changes in postharvest tobacco leaves during curing will provide more information for enhancing the understanding of this biological process and improving crop quality and reducing losses.

iTRAQ (isobaric tags for relative and absolute quantification), a high-throughput proteomic technology, is one platform for comparing changes in the abundance of specific proteins among different samples [8,29,38]. Although new advances have been made in our understanding of pigment metabolism and color change in plant organs [4,5,6,13,23], fewer studies have focused specifically on their molecular mechanisms in postharvest tobacco leaves during curing. In this study, iTRAQ-based proteomic analysis was employed to identify important regulators in pigment metabolism pathways and elucidate the molecular mechanism of pigment metabolism and color change in tobacco leaves during the yellowing stage (0 h, 48 h, 72 h). The results herein provide new insights into the molecular mechanisms involved in pigment metabolism and color change for the future study of postharvest physiological regulatory networks in plants.

## 2. Results

### 2.1. Color and Phenotypic Changes of Tobacco Leaves during Curing

**Figure 1:**
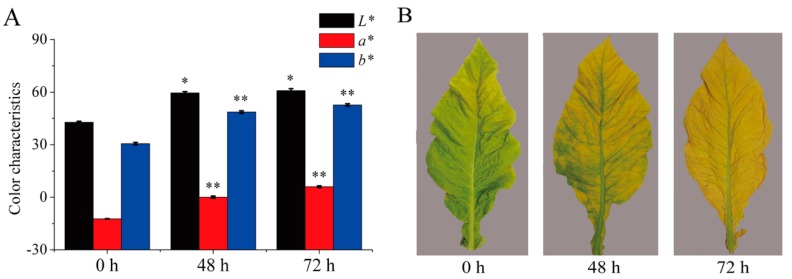
Color and phenotypic changes in tobacco leaves during curing. (**A**) The leaf color values of L*, a*, and b* were determined during curing. Data are shown as the means ± SE, *n* = 30. Asterisks indicate significant differences between the values at 0 h and 48 h or 72 h based on Duncan’s multiple range test in SPSS (* *p* < 0.05, ** *p* < 0.01). (**B**) Representative tobacco leaf phenotypes were documented for each flue-curing stage.

During the curing process, significant differences in the leaf color parameters L*, a*and b* were observed (Figure 1A). Strikingly, the L*, a*, and b* values gradually increased, which were consistent with the changes in the tobacco leaf phenotypes from green to yellow during 0–72 h (Figure 1B).

### 2.2. Ultrastructural Observations of Tobacco Leaves during Curing

**Figure 2:**
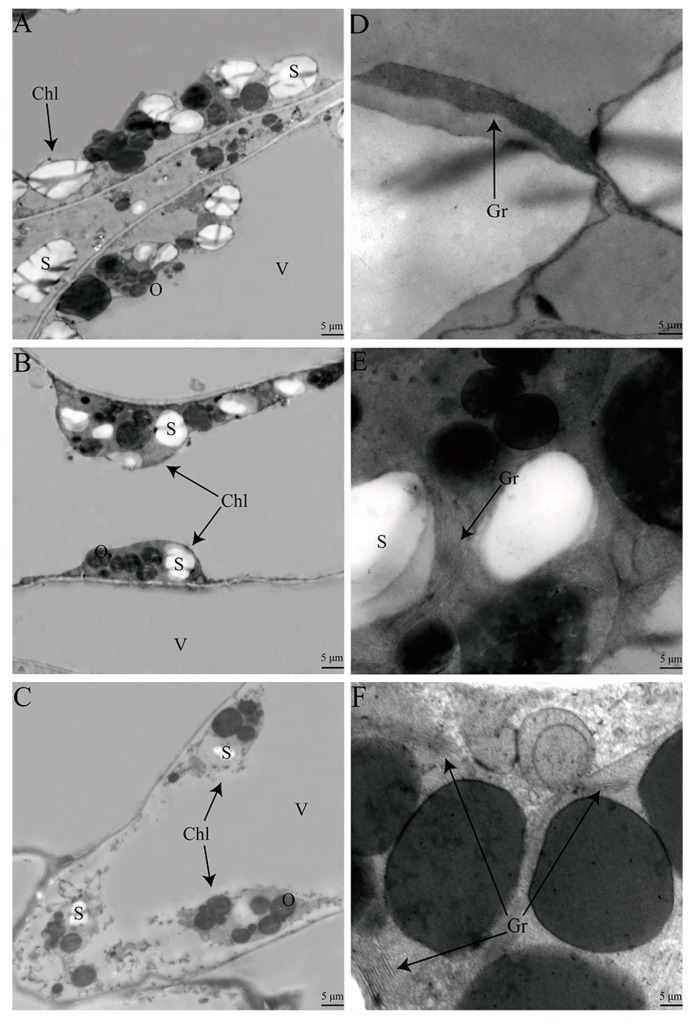
Ultrastructural changes in tobacco leaves during curing. Chloroplasts were gradually disrupted in tobacco leaves cells during curing (**A**, 0 h; **B**, 48 h and **C**, 72 h). Grana thylakoid lamellae were disrupted in tobacco leaves during curing (**D**, 0 h; **E**, 48 h and **F**, 72 h). Chl, chloroplast; Gr, grana thylakoid lamellae; S, starch granule; O, osmiophilic granule; V, vacuole.

Ultrastructural observations indicated that the cell contained relatively intact chloroplast, grana thylakoids, and starch granules at 0 h; however, at 48 h, the chloroplast membranes and grana thylakoid lamellae were severely disrupted (Figure 2). At 72 h, only a few of the chloroplast and grana thylakoid lamellae remained. Ultrastructural observations of the cells showed that the curing process accelerated the chloroplast structural breakdown and promoted the degradation of the pigments in tobacco leaves during curing.

### 2.3. Physiological Attributes of Tobacco Leaves during Curing

**Figure 3:**
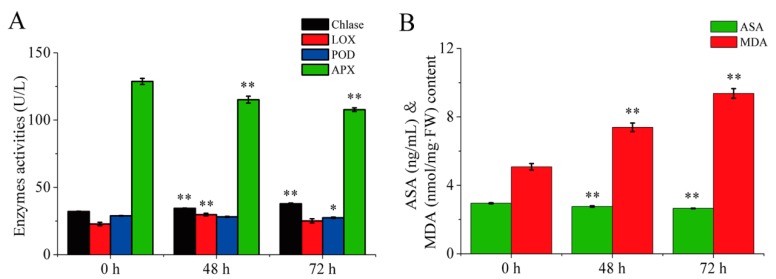
Changes of enzyme activities (**A**, Chlase, LOX, POD, APX) and chemical components (**B**, ASA and MDA) in tobacco leaves during curing. Data are shown as the means ± SE (*n* = 6). Asterisks indicate significant differences between the values at 0 h and 48 h or 72 h based on Duncan’s multiple range test in SPSS (* *p* < 0.05, ** *p* < 0.01). Chlase, chlorophyllase; LOX, lipoxygenase; POD, peroxidase; APX, ascorbate peroxidase; ASA, ascorbic acid; MDA, malondialdehyde.

The plastid pigment concentrations in tobacco leaves during curing were analyzed, as presented in Table 1. Among several carotenoids, the highest levels were displayed by lutein followed by *β*-carotene, while violaxanthin concentration was found higher than that of neoxanthin during 0–72 h. However, the chlorophylls were found to be the most abundant plastid pigments in tobacco leaves at 0 h, followed by the carotenoids. Although the carotenoid and chlorophyll concentrations decreased significantly, the ratio between the carotenoids and chlorophylls significantly increased during curing. This difference may be explained by the observation that the chlorophyll *a* (94.05%) and chlorophyll *b* (87.53%) concentrations and SPAD value (93.37%) decreased at a greater rate than carotenoids, including *β*-carotene (74.35%), lutein (77.56%), violaxanthin (73.71%), and neoxanthin (79.94%) during curing. These results indicated that pigments, particularly chlorophylls, degrade at high levels in tobacco leaves during curing. To confirm that proteins involved in pigment metabolism were modulated in tobacco leaves during 0–72 h, we selected physiological parameters that can be measured using established assays (Figure 3). The Chlase activities and MDA content significantly increased in tobacco leaves during curing. However, ascorbate peroxidase (APX) activities and ascorbic acid (ASA) content significantly decreased in tobacco leaves during 0–72 h. In addition, the LOX activities showed higher at 48 h than that at 0 h and 72 h, and the POD activities in leaves at 0 h was significantly higher than that at 72 h. These findings indicate that change in physiological parameters associated with pigment metabolism and color change is significant in tobacco leaves during different curing stages.

### 2.4. Pigment Degradation Products Analysis in Tobacco Leaves during Curing

For chemometric analysis, the relative concentrations of the 82 volatile components were analyzed using comprehensive two-dimensional gas chromatography time-of-flight mass spectrometry (GC×GC-TOF-MS) system (Supplementary Table S1), including 19 carotenoid metabolites and 1 chlorophyll catabolite (Supplementary Table S2). The total concentration of carotenoid and chlorophyll degradation products in tobacco leaves decreased during 0–48 h and 0–72 h and increased during 48–72 h. It is worth noting that the 6-methyl-5-hepten-2-ol, *β*-ionol, *β*-ionone, and solavetivone detected in the tobacco leaf samples during 0–72 h and the 3-oxo-α-ionol detected in the mature fresh leaves have not been previously reported to be the components in tobacco headspace volatiles. Strikingly, isophorone was found to be the most abundant carotenoid volatile metabolite in tobacco leaves followed by geranylacetone and dihydroactinidiolide during 0–72 h. The levels of six carotenoid metabolites, including 6-methyl-5-hepten-2-ol, linalool, isophorone, megastigmatrienone A, megastigmatrienone B, and solavetivone were decreased during 0–72 h. Conversely, the levels of 3-oxo-α-ionol and 3-hydroxy-*β*-damascone increased during 0–72 h, whereas the remaining metabolites of carotenoid and chlorophyll did not persistently increase or decrease during 0–72 h. These findings indicate that the postharvest tobacco leaves underwent a series of complex physiological and biochemical changes involving pigment metabolism during curing.

### 2.5. Protein Profile Analysis of Tobacco Leaves Using iTRAQ

**Figure 4:**
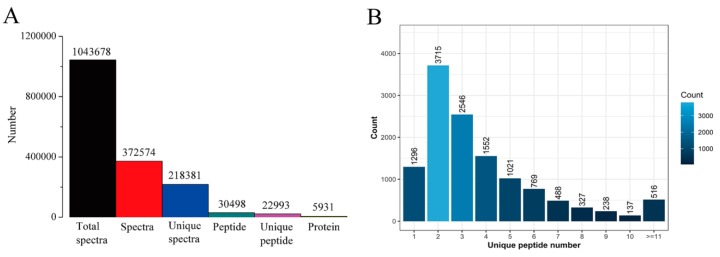
Basic information of iTRAQ output. (**A**) Spectra, peptides, and proteins identified in tobacco leaves. (**B**) Number of peptides that matched proteins.

In order to clarify molecular mechanisms involved in carotenoid and chlorophyll metabolism and color change, data analysis based on the phenotypic, physiological, and chemical changes in tobacco leaves during 0–72 h using iTRAQ was found credible. In total, 1,043,678 spectra were identified from the iTRAQ analysis using the leaf samples at different curing stages as the materials. MASCOT (Modular Approach to Software Construction Operation and Test), a powerful database retrieval software, which can realize the identification from mass spectrometry data to protein, generated a total of 372,574 spectra matched to in silico peptide spectra, 218,381 unique spectra, 30,498 peptides, 22,993 unique peptides, and 5931 proteins from the iTRAQ experiments Run1, Run2, and Run3 (Figure 4A and Supplementary Table S3). In order to obtain the relationship between the spectrum and the peptide segment, the mass spectrum was matched with the theoretical spectrum, where peptide segments were used as the dimension for data processing and calculation (a peptide segment may correspond to more than one spectrum). Among the identified proteins in three technical duplicate experiments, 1296 proteins had 1 identified unique peptide, 3715 had 2, 2564 had 3, 516 had more than 11, and the remainder had 4–10 (Figure 4B). The peptide information validated that many unique peptides are shared with different proteins (the identified peptides were compared with protein databases). The relative molecular mass of identified proteins was mainly distributed at 10~80 kDa, and the proportion (18.06%) of proteins with a relative molecular mass of 30~40 kDa was the highest (Supplementary Figure S1A). A total of 5488 proteins were identified with 0~10% sequence coverage. However, only 1.18% of the proteins were identified with sequence coverage > 20% (Supplementary Figure S1B). The coefficient of variation (CV), defined as the ratio of the standard deviation (SD) to the mean (CV  =  SD/mean) was used to evaluate the reproducibility of protein quantification. The lower the CV, the better the reproducibility. The CV distribution (mean CV: 0.16) in three replicates showed good reproducibility (Supplementary Figure S2).

### 2.6. DEPs Identified and Functional Analysis

**Figure 5:**
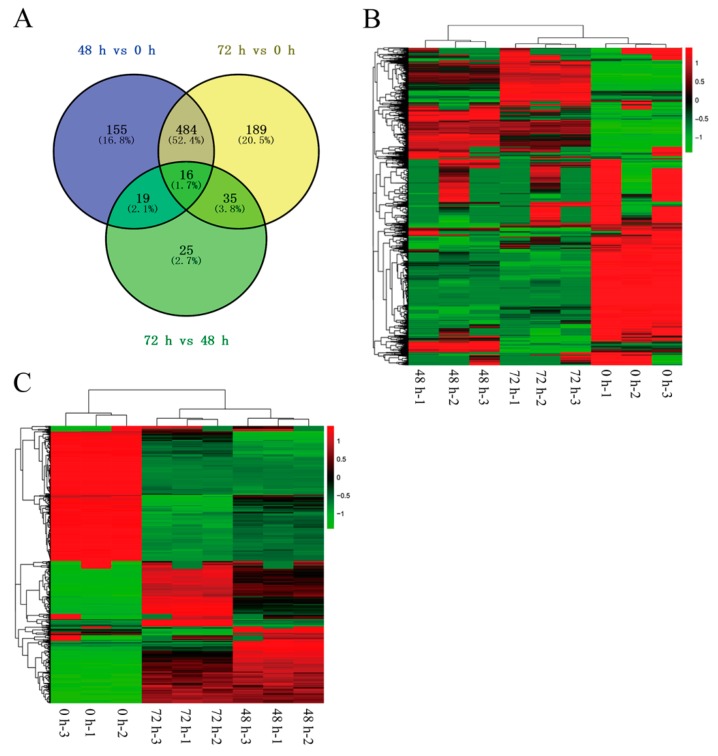
Venn diagram and heatmap representing identified proteins and DEPs from different comparison groups in tobacco leaf samples. (**A**) Total DEPs identified in all tobacco leaf samples; (**B**) Heatmap/hierarchical clustering of all identified proteins; and (**C**) Heatmap/hierarchical clustering of DEPs identified in all tobacco leaf samples. The numbers of DEPs identified from three biological replicates are shown in the different segments (Figure 5A). Red and green indicate higher expression and lower expression, respectively (Figure 5B,C).

The proteins were screened with a fold-change value > 1.5 or < 0.67 and a Q-value of < 0.05. Based on these criteria, 450 (319, 348 and 36) up-regulated proteins, 452 (355, 376 and 59) down-regulated proteins, and 21 commonly expressed (up/down-regulated) proteins with a total of 923 (674, 724 and 95) DEPs in the comparisons of leaves at 48 h and 0 h (“48 h vs. 0 h” hereafter), leaves at 72 h and 0 h (“72 h vs. 0 h” hereafter), and leaves at 72 h and 48 h (“72 h vs. 48 h” hereafter) were identified, respectively (Figure 5A,C and Supplementary Figure S3). The heatmap/hierarchical clustering analysis was conducted for all the identified proteins and DEPs using the pheatmap package in R language. As shown in Figure 5B,C, the identified proteins and DEPs in different leaf samples were easily discriminated, which demonstrated the significant differences in protein levels in tobacco leaves during different curing stages. To further understand their functions, 837 DEPs were annotated on the basis of gene ontology (GO) terms in three categories: Biological process, cellular component, and molecular function (Supplementary Figure S4). In particular, the catalytic activity involved 375, 409, and 48 DEPs in comparisons of 48 h vs. 0 h, 72 h vs. 0 h and 72 h vs. 48 h, and was the most commonly annotated category under the biological process term. In contrast, 389/387, 402/401, and 49/49 DEPs were annotated under cell/cell part in the cellular components term in different comparisons. In the molecular function category, 365/349, 375/357, and 42/40 DEPs were annotated under the cellular process/metabolic process in different comparisons.

According to the biological functional properties, the eukaryotic orthologous groups (KOGs) categories of DEPs are shown in Supplementary Figure S5A–C. Post-translational modification, protein turnover and chaperones (19.90%/16.12%), general function prediction only (13.10%/13.84%), translation, ribosomal structure and biogenesis (11.22%/10.10%), carbohydrate transport and metabolism (9.35%/9.61%), and energy production and conversion (6.12%/8.14%) were the main functional categories identified from the comparisons of 48 h vs. 0 h and 72 h vs. 0 h in tobacco leaves during curing, whereas the main KOGs categories obtained from the comparison of 72 h vs. 48 h were posttranslational modification, protein turnover and chaperones (18.92%), general function prediction only (13.51%), translation, ribosomal structure and biogenesis (12.16%), amino acid transport and metabolism (6.76%), and energy production and conversion (6.76%). Furthermore, the Kyoto Encyclopedia of Genes and Genomes (KEGG) pathway annotation of DEPs are shown in Supplementary Figure S6A–C. The global and overview maps (255, 263, and 28) and carbohydrate metabolism (94, 112, and 14) pathways exhibited more annotated DEPs in different comparisons.

### 2.7. DEPs Involved in Carotenoid and Chlorophyll Metabolism

**Figure 6:**
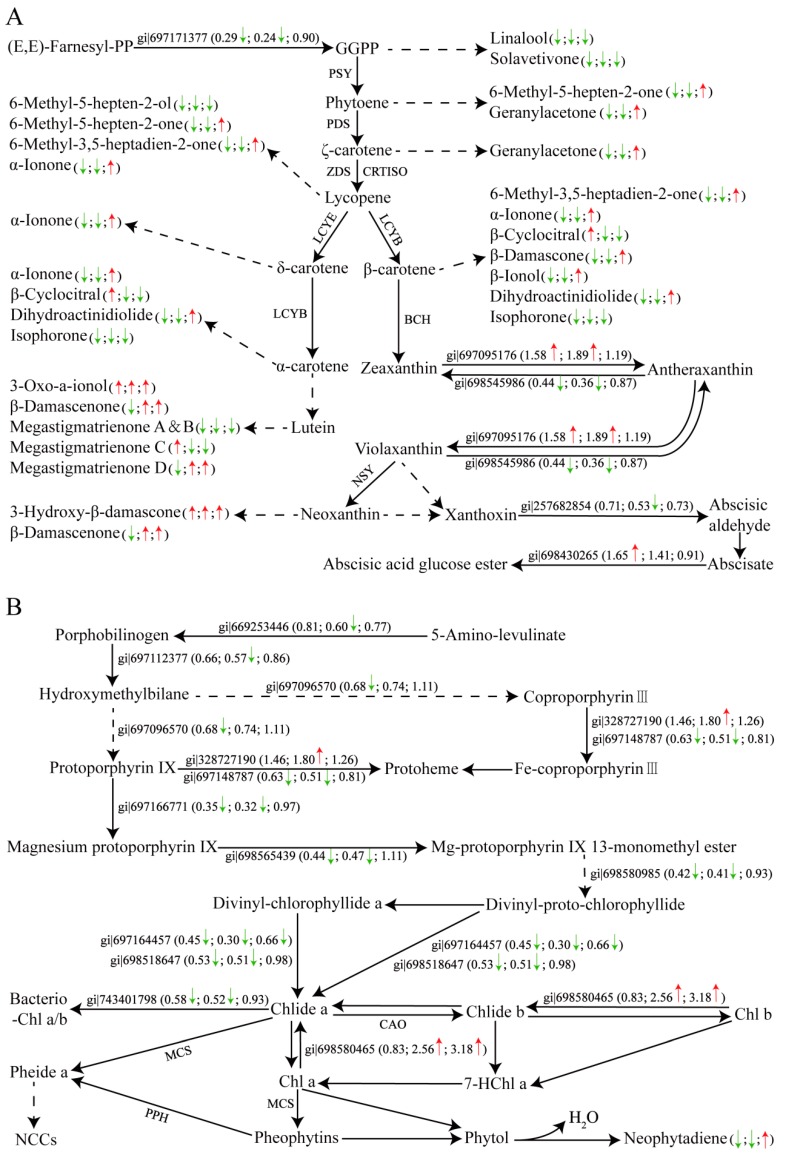
The carotenoid (**A**) and chlorophyll (**B**) metabolic pathway in tobacco leaves during curing. Up-regulated proteins or increased metabolites are marked with upward red arrows, while down-regulated proteins or decreased metabolites are marked with downward green arrows. The numbers represent the fold change. The left arrows or numbers represented the difference of proteins or metabolites during 0–48 h, the middle arrows or numbers indicated the difference during 0–72 h, and the right indicated the difference during 48–72 h. Gi numbers and ratios of the DEPs are shown in Supplementary Table S4. (**A**) GGPP, geranylgeranyl diphosphate; PSY, phytoene synthase; PDS, phytoene desaturase; ZDS, ζ-carotene desaturase; CRTISO, carotenoid isomerase; LCYB, lycopene *β*-cyclase; LCYE, lycopeneε-cyclase; BCH, *β*-carotene hydroxylase; NSY, neoxanthin synthase. (**B**) Chlide a/b, chlorophyllide a/b; MCS, metal-chelating substance; CAO, chlorophyllide-a oxygenase; Pheide a, pheophorbide a; Chl a/b, chlorophyll a/b; 7HChl a, 7-Hydroxy-chlorophyll a; NCCs, non-fluorescent chlorophyll catabolites.

**Figure 7:**
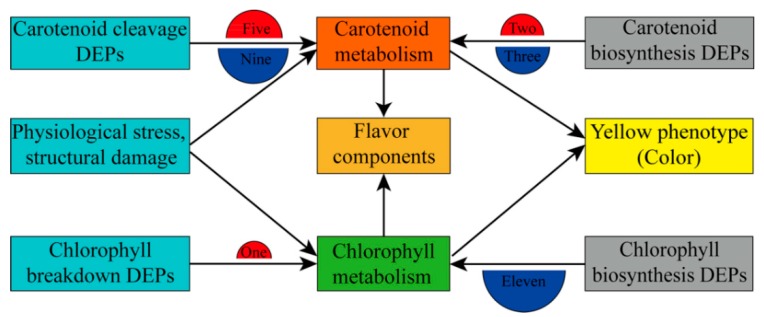
Branching program related to pigment metabolism and color change in tobacco leaves during curing. The semicircle filled with the red color indicated that the DEPs were positive regulators, and the blue color suggested that the DEPs were negative regulators in the pigment biosynthetic and breakdown pathway. The numbers represented the numbers of the total up- and down-regulated proteins in different pathways.

At the post-transcriptional level, 31 DEPs involved in carotenoid and chlorophyll metabolism were identified in tobacco leaves during curing, and the detail of these DEPs and BLAST data are listed in Supplementary Table S4. iTRAQ analysis revealed that among five DEPs in the carotenoid biosynthetic pathway, two were up-regulated during 0–48 h and/or 0–72 h, and three were down-regulated during 0–48 h and/or 0–72 h (Figure 6A). Alternatively, 14 proteins involved in carotenoid degradation were differentially expressed in tobacco leaves during curing. These proteins included 2 LOX proteins and 12 POD proteins. Two LOX proteins were both up-regulated during 0–48 h and 0–72 h. Of the 12 POD proteins, 3 were up-regulated during 0–48 h and/or 0–72 h, and 9 were down-regulated during 0–48 h and/or 0–72 h and/or 48–72 h. The differences in the abundance of these proteins indicated the complex regulatory network involved in carotenoid metabolism in tobacco leaves during curing (Figure 7). A total of 12 DEPs were involved in chlorophyll metabolism in tobacco leaves during curing, including 11 DEPs in the chlorophyll biosynthetic pathway and 1 DEP in the chlorophyll breakdown pathway (Figure 6B). Ten DEPs in the chlorophyll biosynthetic pathway were significantly down-regulated during 0–48 h and/or 0–72 h and/or 48–72 h. In contrast, ferrochelatase isoform I was up-regulated during 0–48 h and 0–72 h in the chlorophyll biosynthetic pathway, and chlorophyllase-1-like isoform X2 (Chlase-1-X2) was significantly up-regulated during 0–72 h and 48–72 h in the chlorophyll breakdown pathway. The results indicated that these DEPs potentially play important roles in chlorophyll metabolism (Figure 7).

### 2.8. Validation of iTRAQ Data by qRT-PCR

**Figure 8:**
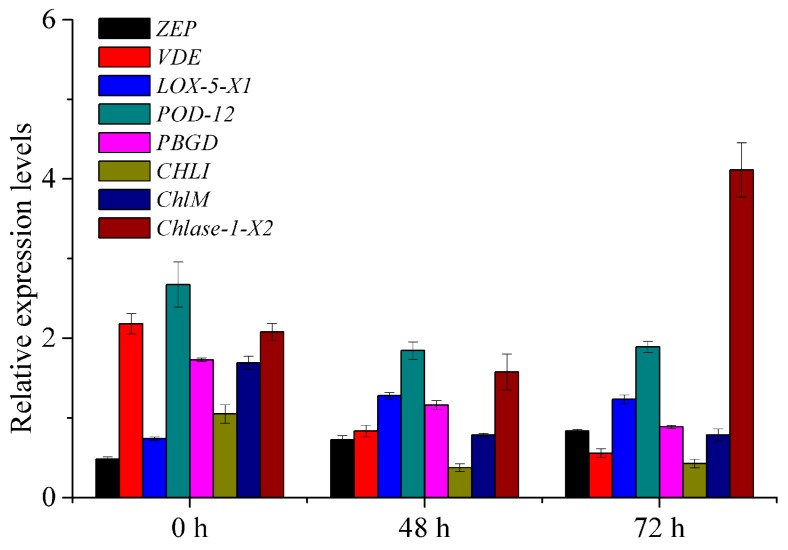
Verification of iTRAQ results by qRT-PCR. Values represent the means ± SE (*n* = 3). *ZEP*, zeaxanthin epoxidase, chloroplastic-like; *VDE*, violaxanthin de-epoxidase, chloroplastic; *LOX-5-X1*, probable linoleate 9S-lipoxygenase 5 isoform X1; *POD-12*, peroxidase 12-like; *PBGD*, porphobilinogen deaminase, chloroplastic-like; *CHLI*, magnesium-chelatase subunit ChlI, chloroplastic; *ChlM*, magnesium protoporphyrin IX methyltransferase, chloroplastic; *Chlase-1-X2*, chlorophyllase-1-like isoform X2.

To provide the accurate data for the molecular mechanisms related to the pigment metabolism and color change in postharvest tobacco leaves during curing, the mRNA expression levels of eight key DEPs were detected by quantitative real-time polymerase chain reaction (qRT-PCR). The results exhibited that qRT-PCR data of eight genes aligned with the iTRAQ results (Figure 8).

## 3. Discussion

Proteomics analysis provides a broad perspective on the process of leaf color change, which prompts us not only to pay attention to the pigment metabolism pathway but also to further screen some key proteins related to the pigment metabolism and color change in tobacco leaves during curing [27,28,29]. Quantitative proteome analysis revealed that hundreds of DEPs were identified in all leaf samples during curing. Although, many DEPs might be associated with pigment metabolism and color change in tobacco leaves during curing, 19 DEPs related to carotenoid metabolism, and 12 DEPs involved in chlorophyll metabolism were selected based on bioinformatics analysis. This analysis helped us to clearly identify DEPs associated with pigment metabolism and color change and the postharvest physiological regulatory networks in tobacco leaves during curing (Figure 7).

### 3.1. Leaf Color Change is Determined by the Carotenoid and Chlorophyll Content

Color change in plants is determined by the content of various plastid pigments, and plant organs are intensely yellow due to the accumulation of the xanthophylls, including lutein, neoxanthin, and violaxanthin [11,25,26]. The green color changes to orange due to the breakdown of chlorophylls and the accumulation of the *β*-carotene in plants [6,25]. Regardless, the concentrations of both carotenoid and chlorophyll significantly decreased in tobacco leaves during curing. Chlorophyll was found to be the most abundant plastid pigment in tobacco leaves at 0 h, but the ratio of carotenoid/chlorophyll of different samples was all larger than or equal to 1.30 during 48–72 h. In addition, the ratios between xanthophylls and *β*-carotene of different samples were all larger than or equal to 1.71 during 0–72 h. Thus, the tobacco leaves showed a green phenotype at 0 h and a yellow phenotype at 48 h and 72 h. Pigment metabolism and their relative contents were responsible for the formation of the yellow phenotype, which was consistent with previous reports [11,30,39]. The data were expressed as the color values of lightness L*, greenness a*, and yellowness b* [2,11,27]. In this study, the change in color was quantified as the increment in the values of L*, a*, and b*, which is associated with the pigment degradation and the increase in the relative concentrations of the carotenoid and the phenotypic change in tobacco leaves during curing.

### 3.2. Effect of Cell Ultrastructure Damage on Pigment Metabolism and Leaf Color Change

The metabolism of chlorophyll and carotenoid occurs in the chloroplast (complex organelle with several distinct sub-organellar compartments to internally sort the proteins) and chromoplast membranes [8,40]. Compared with 0 h, chloroplasts and grana thylakoid lamellae appeared to be more severely damaged in tobacco leaves at 48 h and 72 h, especially at 72 h. Chloroplast structure and functions are plausibly linked to pigment metabolism and leaf color change [8,30]. Structural damage of the chloroplast might accelerate the degradation of the chlorophyll and carotenoid, and alter the proportion of pigment compositions and promote the formation of yellow color in tobacco leaves during curing.

### 3.3. Role of Physiological Parameters in Pigment Metabolism and Leaf Color Change

Chlase, POD, and LOX are all important enzymes involved in pigment metabolism [14,15,19], and APX, ASA, and MDA are significant parameters to deduce the physiological state in plants [41,42]. The increased Chlase activities in tobacco leaves during curing might accelerate the degradation of chlorophyll [17,18]. In contrast, the increased LOX activities in tobacco leaves during 0–48 h might promote the degradation of carotenoid [13,14], but the decreased POD activities during 0–72 h might result in the delayed degradation of carotenoid in tobacco leaves during curing [15]. APX is an important enzyme for detoxification of H_2_O_2_ in plants [43], and ASA is a key substrate for the detoxification of reactive oxygen entities [36]. The decreased APX activities and ASA content in tobacco leaves during 0–72 h might accelerate leaf senescence and promote the degradation of pigment. MDA is a marker for lipid peroxidation and a characteristic of senescence in plants, and the increased MDA content in tobacco leaves during curing might also accelerate leaf senescence and promote the degradation of pigment, which is consistent with the results of leaf senescence in tomato plants [42]. Following the progressive stress of physiology and catalysis of the enzymes, the pigment content remarkably decreased during curing, especially chlorophyll content, and the leaves kept a yellow phenotype for 72 h.

### 3.4. Role of DEPs in Carotenoid and Chlorophyll Metabolism and Color Change

In this study, 31 DEPs involved in carotenoid and chlorophyll metabolism were identified in tobacco leaves during curing. Although, these DEPs in the pigment metabolic pathway were mainly responsible for the change of pigment content and leaf color, the carotenoid cleavage, and chlorophyll breakdown were the primary biological processes in postharvest plant leaves during curing [16,23,44]. In the present study, 5 DEPs involved in carotenoid biosynthesis and 14 DEPs related to carotenoid cleavage were identified in tobacco leaves during curing. In the carotenoid biosynthetic pathway, geranylgeranyl pyrophosphate synthase, chloroplastic-like (GGPPS) was down-regulated in tobacco leaves during curing, which catalyzes the conversion of (E, E)-farnesyl diphosphate (FPP) into geranylgeranyl diphosphate (GGPP). In plants, FPP and GGPP are isoprenoid precursors necessary for carotenoid biosynthesis. The down-regulated GGPPS in tobacco leaves during 0–48 h and 0–72 h might reduce the content of carotenoids, and it ultimately leads to the decreased content of their degradation products, such as linalool, solavetivone, 6-methyl-5-hepten-2-ol, 6-methyl-5-hepten-2-one, 6-methyl-3,5-heptadien-2-one, *β*-ionone, and isophorone.

In addition, zeaxanthin epoxidase, chloroplastic-like (ZEP) was significantly up-regulated, and VDE was significantly down-regulated in tobacco leaves during 0–48 h and 0–72 h. VDE is a member of a group of proteins known as lipocalins that bind and transport small hydrophobic molecules [7]. Zeaxanthin is epoxidized by ZEP, finally yielding violaxanthin, however, this epoxidation is reversible with the effect of VDE [11]. When zeaxanthin synthesis is inhibited by VDE, violaxanthin might be catalyzed by neoxanthin synthase (NSY) to yield neoxanthin. Then neoxanthin could be converted into 3-hydroxy-*β*-damascone and *β*-damascenone under the catalysis of carotenoid cleavage enzymes, resulting in the increase in their concentrations in tobacco leaves during 0–72 h. Furthermore, the violaxanthin concentration was higher than that of neoxanthin during 0–72 h, which might be closely associated with the up-regulated ZEP and down-regulated VDE.

In the carotenoid biosynthetic pathway, unnamed protein product protein was significantly down-regulated in tobacco leaves during 0–72 h, while scopoletin glucosyltransferase-like protein was significantly up-regulated during 0–48 h. Both of them were involved in ABA biosynthesis in tobacco leaves during curing. The down-regulated unnamed protein product protein might not be helpful for the biosynthetic conversion of xanthoxin into ABA, whereas the up-regulated scopoletin glucosyltransferase-like protein might be conducive to ABA biosynthesis and finally lead to the decreased content of carotenes. Alternatively, it is worth emphasizing that CCDs are known to be important for cleaving carotenoid compounds and forming important flavor and fragrance volatiles or their apocarotenoids [14,39,45]. However, none of them showed a significant up- or down-regulation in different comparisons. Thereby, we speculate that CCDs might not be crucial for carotenoid metabolism in tobacco leaves during 0–72 h. Moreover, LOX and POD are important enzymes involved in the cleavage of carotenoids [13,14,15]. Two up-regulated LOX proteins might accelerate the degradation of carotenoids and keep them at low levels in tobacco leaves during curing. Three POD proteins were up-regulated, but the other nine POD-related proteins were down-regulated in tobacco leaves during curing. These up-regulated POD proteins might accelerate the degradation of carotenoids, but down-regulated POD proteins might not be conducive to carotenoid cleavage and forming important flavor. In addition, 19 carotenoid metabolites showed 6 changing trends with increased and/or decreased relative concentrations in different curing stages. These results suggested that carotenoid metabolism was involved in a complex regulatory network.

In contrast, a total of 12 DEPs were involved in chlorophyll metabolism in tobacco leaves during curing, including 1 up-regulated and 10 down-regulated DEPs in the chlorophyll biosynthetic pathway and 1 up-regulated DEP in the chlorophyll breakdown pathway. The down-regulated delta-aminolevulinic acid dehydratase, porphobilinogen deaminase, chloroplastic-like, protoporphyrinogen oxidase, chloroplastic, magnesium-chelatase subunit ChlI, chloroplastic isoform X1, magnesium protoporphyrin IX methyltransferase, chloroplastic, magnesium-protoporphyrin IX monomethyl ester [oxidative] cyclase, chloroplastic, uncharacterized protein ycf39 isoform X2, protein TIC 62, chloroplastic isoform X3 and geranylgeranyl reductase in the chlorophyll biosynthetic pathway inhibited chlorophyll biosynthesis and indirectly reduced chlorophyll content in tobacco leaves during curing, which is consistent with previous reports related to color regulation in plants [8,27,29]. Additionally, two DEPs were identified in the chlorophyll biosynthesis shunt related to protoheme. The ferrochelatase isoform I protein was significantly up-regulated during 0–72 h, while ferrochelatase-2, chloroplastic isoform X1 protein was significantly down-regulated during 0–48 h and 0–72 h, which might indirectly regulate chlorophyll metabolism via an effect targeted on the coproporphyrin III and protoporphyrin IX. The results suggested that these DEPs were negative regulators of chlorophyll biosynthesis in tobacco leaves during curing.

Alternatively, it is worth emphasizing that Chlase-1-X2 catalyzes the conversion of chlorophyll into chlorophyllide and phytol and is thought to be a key rate-limiting step in the chlorophyll breakdown pathway [19,46]. Subsequently, neophytadiene is formed via the dehydration of phytol. The Chlase-1-X2 was significantly up-regulated during 0–72 h and 48–72 h, which accelerated chlorophyll degradation in tobacco leaves during curing. However, the Chlase-1-X2 was down-regulated (mean ratio = 0.83) in tobacco leaves during 0–48 h. The relative concentrations of neophytadiene decreased markedly during 0–48 h and increased significantly during 48–72 h, which indicated a positive correlation between neophytadiene and Chlase-1-X2 protein. Thus, we inferred that Chlase-1-X2 played a leading role in chlorophyll breakdown in tobacco leaves during curing.

## 4. Materials and Methods

### 4.1. Plant Material and Sampling

Tobacco cultivar “Bi’na1” was obtained from the Guizhou Academy of Tobacco Science, China. Tobacco plants were grown in Fuquan City, Guizhou Province, Southwest China. Plants were cultivated based on the local production standard to produce high-quality tobacco leaves. For the flue-curing experiment, uniform mature leaves from the middle parts of the tobacco plants (at the 10^th^ leaf position; from the bottom to approximately 60 cm in height) in individually labeled plants were harvested 80 days after field transplantation. The flue-curing barns were designed by the Guizhou Academy of Tobacco Science based on the Technique Standard of Bulk Curing Barns (Probative) Castigatory Version No. [2008]575. The curing schedule was executed following the Code of Practice for Tobacco Curing by Loose-leaves (YC/T 457-2013) (Supplementary Figure S7). It is worth noting that an intelligent curing system (DDMB06YS) was adopted, which can automatically control the dry bulb and wet bulb temperatures during curing.

For each experiment, tobacco leaves were collected at 3 phases of flue-curing, i.e., 0 h, 48 h, and 72 h during the yellowing stage. Leaf samples of 0 h were collected from labeled plants in the fields before flue-curing. At 48 h (from the beginning of the curing process), approximately 80% of the leaf area turned yellow in the middle of the yellowing stage (dry bulb temperature 38 °C and wet bulb temperature 35~36 °C). At 72 h, the tobacco leaf had the yellow laminae and green midribs indicating the end of the yellowing stage (dry bulb temperature 42 °C and wet bulb temperature 33~34 °C). The samples were divided into two duplicates at each stage during curing; one was used directly to determine the color parameters, cell ultrastructure, and pigment content, and the other one was frozen in liquid nitrogen immediately to further measure the carotenoid composition and degradation products, physiological parameters and the DEPs involved in pigment metabolism. Three independent curing experiments were performed.

### 4.2. Color Analysis

The leaf color was determined at 0 h, 48 h, and 72 h using a Minolta Chroma Meter CR-10 (Konica Minolta Sensing, Inc., Japan) calibrated previously with a white standard tile by taking 6 measurements per leaf in the equatorial region. The data were expressed as the color values of lightness (L* = measures light reflected), redness (a* = measures positive red and negative green), yellowness (b* = measures positive yellow and negative blue). Thirty leaves were used for these determinations at each yellowing stage during curing.

### 4.3. Ultrastructural Observation

Sample sections of 1 mm^2^ (2 cm distance from the midrib) were excised from the middle portion of the labeled leaves. Ultrastructural changes were studied by observing ultrathin sections of leaf palisade tissue at 0 h, 48 h, and 72 h using a Hitachi H-600 electron microscope (Kyoto, Japan) [47].

### 4.4. Physiological Measurements

Approximately 0.1 g of fresh tissue was immersed in 95% ethanol for 24 h in the absence of light. The absorbance of the extracts was measured using a UV-1800 ultraviolet/visible spectrophotometer (Shimadzu, Kyoto, Japan) at wavelengths of 470, 649, and 665 nm. Chlorophyll *a*, chlorophyll *b*, and total carotenoid concentrations were calculated as previously described [48]. The frozen leaf samples were ground into a fine powder using liquid nitrogen with a mortar and pestle, then freeze-dried. The *β*-carotene, lutein, neoxanthin, and violaxanthin were quantified via HPLC, as described previously [49]. Moreover, the chlorophyll content, as expressed by the SPAD value, was measured using a Chlorophyll Meter (Model SPAD-502, Tokyo, Japan). Thirty leaves were measured by taking 6 measurements per leaf in the equatorial region. The leaf samples were analyzed for Chlase, LOX, POD, APX, ASA activities, and malondialdehyde (MDA) content. Plant enzyme-linked immunosorbent assay kits were purchased from the Shanghai Jianglai Bio-Technology Co., Ltd. (Shanghai, China) to measure Chlase and LOX activity, and from the Nanjing Jiancheng Bioengineering Institute (Nanjing, China) to determine the APX and POD activities, along with ASA and MDA content.

### 4.5. Pigment Degradation Products Analysis

Carotenoid and chlorophyll degradation products were determined using qualitative and quantitative methods in postharvest tobacco leaves during curing. Freeze-dried tobacco leaf samples were previously treated using headspace solid-phase micro-extraction (HS-SPME) and analyzed using a GC×GC-TOF-MS [50].

### 4.6. Protein Extraction

Total proteins were extracted from leaf tissue at 0 h, 48 h, and 72 h during curing, as previously described [51]. The samples were transferred to a 2 mL centrifuge tube, and 5% cross-linked polyvinylpyrrolidone (PVPP) powder and homogenization lysis buffer (7 M urea, 2 M thiourea, 4% 3-[(3-cholamidopropyl) dimethylammonio]-1-propanesulfonate [CHAPS], 40 mM Tris-HCl, pH 8.5) were added. A grinder (power is 60 HZ, time is 2 min) was used to break the tissues, then 2 × volume of Tris-saturated phenol was added and shaken for 15 min. After centrifugation (25,000× *g* for 15 min at 4 °C), the upper phenol phase was taken into a 10 mL centrifuge tube, and 5 × volume of 0.1 M cold ammonium acetate/methanol and 10 mM dithiothreitol (DTT; final concentration) were added, then placed at −20 °C for 2 h. These steps were repeated twice. Then, 1 mL of cold acetone was added and again placed at −20 °C for 30 min. The supernatant was discarded after centrifugation, and this step was repeated once. Air-dry precipitation, 1XCocktail was added with SDS L3 and ethylene diamine tetra acetic acid (EDTA), then 10 mM DTT was added after putting on ice for 5 min. Protein was solubilized using a grinder and centrifuged, and then the supernatant was discarded and put into a water bath for 1 h at 56 °C after adding 10 mM DTT. Afterward, 55 mM iodoacetamide (IAM) was added and placed in a dark room for 45 min. 1 mL cold acetone was added, and placed at −20 °C for 2 h, then centrifuged. The steps of protein solubilization and centrifugation were repeated. The protein concentration was determined by the Bradford assay using bovine serum albumin (BSA) as a standard [52]. The samples were kept at –80 °C for further analysis.

### 4.7. iTRAQ Labeling and Strong Cation Exchange (SCX) Fractionation

Proteins were digested using trypsin gold (Promega, Madison, WI, USA) with the ratio of protein:trypsin = 30:1 at 37 °C for 16 h. Peptides were processed according to the manufacturer’s protocol for an 8-plex iTRAQ reagent (Applied Biosystems, Foster City, CA, USA). Three protein samples were labeled with the iTRAQ tags as follows: 0 h (115 tag), 48 h (119 tag), and 72 h (121 tag). SCX chromatography was performed using an LC-20AB HPLC pump system (Shimadzu, Kyoto, Japan). The iTRAQ-labeled peptide mixtures were reconstituted with 4 mL buffer A (25 mM NaH_2_PO_4_ in 25% ACN, pH 2.7) and loaded onto a 4.6 mm × 250 mm Ultremex SCX column containing 5 μm particles (Phenomenex). The peptides were eluted at a flow rate of 1 mL/min with a gradient of buffer A for 10 min, 5–60% buffer B (25 mM NaH_2_PO_4_, 1 M KCl in 25% ACN, pH 2.7) for 27 min, 60%\–100% buffer B for 1 min.

### 4.8. LC-ESI-MS/MS Analysis

Each fraction was resuspended in buffer A (2% acetonitrile, 0.1% formic acid) and centrifuged at 20,000× *g* for 10 min. The samples were loaded at 8 μL min^−1^ for 4 min, and the 44 min gradient was then run at 300 nL min^−1^ starting from 2% to 35% B (98% acetonitrile, 0.1% formic acid), followed by a 2 min linear gradient to 80%, and maintenance at 80% B for 4 min, and finally a return to 5% in 1 min. The peptides were subjected to nanoelectrospray ionization followed by tandem mass spectrometry (MS/MS) in a QEXACTIVE (Thermo Fisher Scientific, San Jose, CA, USA) coupled online to the HPLC for data-dependent acquisition (DDA) mode detection. The main parameters were set: The ion source voltage was set to 1.6 kV; the MS1 scan range was 350~1600 m/z; the resolution was set to 70,000; the MS2 starting m/z was fixed at 100; the resolution was 17,500. The screening conditions for the secondary fragmentation were: Charge 2+ to 7+, and the top 20 parent ions with the peak intensity exceeding 10,000. The ion fragmentation mode was the high-energy collision dissociation (HCD), and the fragment ions were detected in Orbitrap. The dynamic exclusion time was set to 15 s. Automatic gain control (AGC) was set to: MS1 3E6, MS2 1E5.

### 4.9. iTRAQ protein Identification and Quantification

The MASCOT search engine (Matrix Science, London, UK; version 2.3.02) was used to simultaneously identify and quantify proteins against the *Nicotiana tabacum* database (http://www.ncbi.nlm.nih.gov/protein?term=txid4085[Organism]; 85,194 entries). For protein identification, a mass tolerance of 20 Da (ppm) was permitted for intact peptide masses, and a mass tolerance of 0.05 Da was permitted for fragmented ions; there was an allowance for one missed cleavage in the trypsin digests. Oxidation (M) and iTRAQ8plex (Y) represent variable modifications, and carbamidomethyl (C), iTRAQ8plex (N-term) and iTRAQ8plex (K) represent fixed modifications. All unique peptides (at least one unique spectrum) were permitted for protein quantitation. An automated software called IQuant [53] was employed for quantitatively analyzing the labeled peptides with isobaric tags. It integrates Mascot Percolator [54], a well-performing machine learning method for rescoring database search results, to provide reliable significance measures. To assess the confidence of peptides, the peptide spectral matches (PSMs) were pre-filtered at 1% PSM-level false discovery rate (FDR). In order to control the rate of false-positive at the protein level, a protein FDR at 1%, which was based on “picked” protein FDR strategy [55], would also be estimated after protein inference (protein-level FDR ≤ 0.01). DEPs were required to satisfy these conditions for identification: Confident protein identification involved at least one unique peptide, changes of greater than 1.5-fold or less than 0.67-fold, and Q-values less than 0.05 in at least 2 replicate experiments. The quantitative protein ratios were then weighted and normalized by the median ratio in MASCOT.

### 4.10. Bioinformatics Analysis

The GO database (http://en.wikipedia.org/wiki/Gene_Ontology) represents an international standardization of gene functional classification systems. The KOGs database (https://www.ncbi.nlm.nih.gov/pubmed/14759257) was used for orthologous protein classification. The pathways were used as queries to search the KEGG pathway database (http://www.genome.jp/kegg/pathway.html). Heatmap/hierarchical clustering of DEPs was conducted by pheatmap package in R language. The mass spectrometry proteomics data have been deposited to the iProX data repository (National Center for Protein Sciences, Beijing, China) with the dataset identifier IPX0001410001 (https://www.iprox.org/).

### 4.11. RNA Extraction and qRT-PCR Analysis

Total RNA was extracted from tobacco leaf samples by TRIzol reagent (Invitrogen), and cDNA was reverse transcribed from 1 μg of total RNA using PrimeScript™ RT Reagent Kit (TaKaRa), according to the manufacturer’s instructions. qRT-PCR was performed using the iQ™5 real-time PCR detection system (Bio-Rad, USA) with the following conditions: 95 °C for 15 s, followed by 40 cycles of 95 °C for 15 s, 60 °C for 30 s, and 72 °C for 30 s. The tobacco *β-actin* gene was used as an endogenous control. The transcript levels of genes were calculated according to the 2^-ΔΔCt^ method [56]. Experiments were performed in triplicate for each treatment. Primer sequences are listed in Supplementary Table S5.

### 4.12. Data Analysis

Data were analyzed statistically using Duncan’s Multiple Range Test with SPSS version 16.0 (SPSS, Chicago, IL, USA). All the photographs and figures were processed and analyzed using Adobe Illustrator CS5 (Adobe Systems Inc., San Francisco, CA, USA) or Origin 8.0 software (Origin lab, Corp., Northampton, MA, USA).

## 5. Conclusions

There was a significant decrease in the content of chlorophyll than carotenoid in tobacco leaves during curing, which was not only associated with the complex regulation of DEPs in carotenoid metabolism, but also correlated with DEPs playing a negative role in chlorophyll biosynthesis and a positive role in chlorophyll breakdown. The total concentration of carotenoid and chlorophyll degradation products in tobacco leaves decreased during 0–48 h and 0–72 h and increased during 48–72 h, which was the result of the combined action of DEPs in the pigment metabolic pathway, especially in the breakdown pathway. These DEPs delayed the degradation of xanthophylls and accelerated the breakdown of chlorophylls, promoting the formation of yellow color during 0–72 h. In particular, the up-regulation of the Chlase-1-X2 was the key protein regulatory mechanism responsible for chlorophyll metabolism and color change. In the future, we will attempt to carry out further research to elucidate the regulatory factors (e.g., environmental and genetic factors) that regulate the pigment metabolic flow and color change in postharvest tobacco leaves during curing. All these findings provide useful molecular information for a better understanding of the complicated postharvest physiological regulatory networks and the molecular mechanisms involved in pigment metabolism and color change in plants.

## Figures and Tables

**Table 1:** Pigment content changes in tobacco leaves during curing.

Concentration	Curing Time (h)		
	0	48	72
*β*-carotene (*μ*g·g_DM_^‒1^)	275.37 ± 7.32	151.19 ± 3.92**	70.63 ± 1.20**
Lutein (*μ*g·g_DM_^‒1^)	384.29 ± 5.02	213.85 ±6.64**	86.23 ± 1.93**
Violaxanthin (*μ*g·g_DM_^‒1^)	99.73 ± 2.98	46.49 ± 2.55**	26.22 ± 0.62**
Neoxanthin (*μ*g·g_DM_^‒1^)	39.72 ± 1.53	14.85 ± 0.44**	7.97 ± 0.28**
Chlorophyll a (mg·g_FM_^‒1^)	0.71 ± 0.02	0.10 ± 0.01**	0.04 ± 0.01**
Chlorophyll b (mg·g_FM_^‒1^)	0.33 ± 0.01	0.07 ± 0.01**	0.04 ± 0.01**
SPAD value	21.87 ± 0.63	5.36 ± 0.39**	1.45 ± 0.23**
Carotenoids/Chlorophylls	0.29 ± 0.01	1.30 ± 0.06**	2.22 ± 0.14**
Xanthophylls/*β*-carotene	1.91 ± 0.06	1.84 ± 0.07	1.71 ± 0.03*

Data are shown as the means ± SEs (*n* = 7), except for the relative chlorophyll content (SPAD; Soil Plant Analysis Development) value (*n* = 30). Asterisks indicate significant differences between the values at 0 h and 48 h or 72 h based on Duncan’s multiple range test in SPSS (* *p* < 0.05, ** *p* < 0.01). Xanthophylls include neoxanthin, violaxanthin, and lutein. DM, dry mass; FM, fresh mass.

## Supplementary Materials

Supplementary materials can be found at https://www.mdpi.com/1422-0067/21/7/2394/s1. Supplementary Figure S1. Protein mass distribution in tobacco leaves (**A**) and coverage of the proteins by the identified peptides (**B**). Supplementary Figure S2. The distribution of coefficient of variation in three replicates. (**A**) Replicate 1 of different leaf samples; (**B**) replicate 2 of different leaf samples; (**C**) replicate 3 of different leaf samples. Supplementary Figure S3. Volcano plots and Venn diagram of differentially expressed proteins in three replicates. (**A**) 48 h vs 0 h; (**B**) 72 h vs 0 h; (**C**) 72 h vs 48 h; (**D**) up-regulated proteins; (**E**) down-regulated proteins Supplementary Figure S4. Barplot of the Gene Ontology analysis of DEPs obtained from different comparisons in tobacco leaf samples. (**A**) 48 h vs 0 h; (**B**) 72 h vs 0 h; (**C**) 72 h vs 48 h. Supplementary Figure S5. KOGs categories of DEPs obtained from different comparisons in tobacco leaf samples. (**A**) 48 h vs 0 h; (**B**) 72 h vs 0 h; (**C**) 72 h vs 48 h. Supplementary Figure S6. Barplots of KEGG pathway analysis of DEPs obtained from different comparisons in tobacco leaf samples. (**A**) 48 h vs 0 h; (**B**) 72 h vs 0 h; (**C**) 72 h vs 48 h. Supplementary Figure S7. Curing schedule of tobacco leaves. Supplementary Table S1. The relative concentrations of the 82 volatile components in tobacco leaves during curing (ng g^−1^). Supplementary Table S2. Plastid pigment metabolites in tobacco leaves during curing. Supplementary Table S3. An overview of protein identification in tobacco leaves during curing. Supplementary Table S4. DEPs involved in pigment metabolism and color change in tobacco leaves during curing. Supplementary Table S5. Primers used for qRT-PCR.
