# Supplementary material for: Comparative Proteomic Analysis by iTRAQ Reveals that Plastid Pigment Metabolism Contributes to Leaf Color Changes in Tobacco (Nicotiana tabacum) during Curing
Source: Int J Mol Sci. 2020 Mar 31;21(7):2394. doi: 10.3390/ijms21072394 (PMC7178154; doi:10.3390/ijms21072394)
Supplement: Supplementary file 1 [file ijms-21-02394-s001.zip › ijms-735811-supplementary.docx]

**Comparative proteomic analysis by iTRAQ reveals that** **plastid pigment metabolism contributes to leaf color changes in tobacco (*Nicotiana tabacum*) during curing**

Shengjiang Wu^1,2^, Yushuang Guo^2^, Muhammad Faheem Adil^4^, Shafaque Sehar^4^, Bin Cai^2^, Zhangmin Xiang^2^, Yonggao Tu^2^, Degang Zhao^1,3^*, Imran Haider Shamsi^4^*

^1^State Key Laboratory Breeding Base of Green Pesticide and Agricultural Bioengineering, Center for Research & Development of Fine Chemicals, The Key Laboratory of Plant Resources Conservation and Germplasm Innovation in Mountainous Region (Ministry of Education), Guizhou University, Guiyang 550025, P.R. China

^2^Guizhou Academy of Tobacco Science, Key Laboratory of Molecular Genetics/Upland Flue-cured Tobacco Quality and Ecology Key Laboratory, CNTC, Guiyang 550081, P.R. China

^3^Guizhou Academy of Agricultural Sciences, Guiyang 550006, P.R. China

^4^Department of Agronomy, College of Agriculture and Biotechnology, Key Laboratory of Crop Germplasm Resource, Zhejiang University, Hangzhou, 310058, P.R. China

*Corresponding author’s e-mail: [dgzhao@gzu.edu.cn](mailto:dgzhao@gzu.edu.cn), drimran@zju.edu.cn

**Table S1.** The relative concentrations of the 82 volatile components in tobacco leaves during curing (ng g^-1^).

| BlobID | Compound Name | 0h | 0h | 0h | 0h | 0h | 0h | 48h | 48h | 48h | 48h | 48h | 48h | 72h | 72h | 72h | 72h | 72h | 72h |
| --- | --- | --- | --- | --- | --- | --- | --- | --- | --- | --- | --- | --- | --- | --- | --- | --- | --- | --- | --- |
| 1 | Acetic acid | 127.41 | 127.22 | 100.86 | 113.75 | 118.65 | 121.56 | 123.76 | 115.87 | 51.95 | 57.64 | 37.25 | 65.90 | 54.76 | 58.39 | 39.65 | 62.40 | 51.86 | 34.27 |
| 2 | Pyridine | 15.39 | 11.85 | 10.53 | 11.64 | 12.72 | 11.55 | 13.79 | 12.49 | 5.99 | 10.11 | 9.06 | 6.48 | 9.50 | 7.40 | 10.07 | 11.50 | 10.03 | 9.70 |
| 3 | 2,3-Butanediol | 26.65 | 36.78 | 25.20 | 27.81 | 29.69 | 30.49 | 28.78 | 24.98 | 16.29 | 14.54 | 10.54 | 12.38 | 9.27 | 15.39 | 13.42 | 19.00 | 8.73 | 6.04 |
| 4 | Butyric acid | 135.22 | 182.76 | 120.04 | 144.99 | 146.90 | 146.39 | 147.76 | 160.84 | 35.95 | 37.06 | 26.95 | 32.24 | 28.46 | 29.35 | 20.02 | 43.32 | 13.97 | 10.47 |
| 5 | 1,3-Butanediol | 221.65 | 293.31 | 207.10 | 233.40 | 243.25 | 245.53 | 244.90 | 267.91 | 151.99 | 150.30 | 117.00 | 135.69 | 109.51 | 128.49 | 147.83 | 202.62 | 95.91 | 70.84 |
| 6 | Furfural | 11.21 | 9.27 | 9.55 | 6.28 | 11.28 | 9.39 | 13.81 | 11.23 | 3.69 | 5.76 | 4.46 | 4.61 | 3.76 | 3.44 | 3.45 | 4.50 | 6.76 | 5.40 |
| 7 | 2-Hexenal | 18.13 | 16.57 | 15.93 | 14.29 | 18.41 | 16.45 | 16.46 | 17.33 | 21.53 | 16.71 | 15.01 | 31.39 | 34.42 | 28.38 | 34.59 | 41.42 | 34.05 | 31.82 |
| 8 | 3-Hexen-1-ol | 38.12 | 37.55 | 31.98 | 31.84 | 36.61 | 36.50 | 38.52 | 34.60 | 4.51 | 5.04 | 3.94 | 5.35 | 5.65 | 4.59 | 5.79 | 6.32 | 5.63 | 5.18 |
| 9 | Hexanoic acid | 40.43 | 51.47 | 39.33 | 48.49 | 42.21 | 46.39 | 49.11 | 50.12 | 23.31 | 33.29 | 21.73 | 28.39 | 36.32 | 28.43 | 35.41 | 36.15 | 17.84 | 13.01 |
| 10 | Benzaldehyde | 170.28 | 177.51 | 193.37 | 180.84 | 175.05 | 180.44 | 185.26 | 169.40 | 129.62 | 136.90 | 118.98 | 121.24 | 111.94 | 126.49 | 128.45 | 139.19 | 167.23 | 134.00 |
| 11 | 6-methyl-5-Hepten-2-one | 9.79 | 9.20 | 8.78 | 9.13 | 8.35 | 10.09 | 9.22 | 9.51 | 5.51 | 5.98 | 5.65 | 4.52 | 0.90 | 5.46 | 4.83 | 4.68 | 5.23 | 4.39 |
| 12 | Phenol | 120.46 | 146.30 | 168.20 | 150.46 | 118.59 | 135.70 | 165.64 | 146.50 | 72.16 | 88.26 | 71.79 | 74.59 | 72.06 | 78.94 | 82.92 | 101.90 | 66.81 | 50.68 |
| 13 | 3-Octanone | 5.00 | 5.46 | 5.66 | 6.55 | 4.32 | 3.49 | 5.04 | 6.56 | 1.66 | 2.46 | 1.98 | 2.38 | 2.52 | 2.14 | 2.91 | 3.40 | 1.46 | 1.19 |
| 14 | 3-Pyridinecarboxaldehyde | 8.17 | 8.30 | 11.21 | 7.18 | 11.85 | 8.40 | 9.87 | 9.24 | 7.83 | 7.75 | 7.29 | 6.38 | 5.78 | 6.90 | 7.45 | 7.57 | 6.50 | 7.93 |
| 15 | 2,4-Hexadienal | 12.78 | 4.95 | 5.66 | 4.78 | 8.18 | 6.49 | 9.46 | 8.39 | 7.75 | 14.43 | 12.29 | 8.75 | 14.29 | 11.24 | 13.07 | 14.83 | 9.34 | 6.20 |
| 16 | 6-methyl-5-Hepten-2-ol | 2.06 | 2.44 | 1.60 | 1.82 | 2.12 | 2.05 | 1.99 | 2.16 | 1.18 | 2.05 | 1.32 | 1.81 | 1.63 | 2.08 | 1.76 | 1.87 | 1.58 | 1.31 |
| 17 | 3,4-dimethyl-2,5-Furandione | 1.56 | 1.73 | 1.53 | 1.73 | 1.60 | 1.65 | 1.53 | 1.46 | 0.89 | 1.13 | 0.91 | 1.24 | 0.93 | 0.85 | 0.39 | 1.45 | 0.94 | 0.66 |
| 18 | 2-Ethyl-1-hexanol | 85.52 | 102.15 | 78.22 | 105.69 | 86.01 | 95.25 | 73.12 | 84.24 | 89.82 | 123.91 | 93.04 | 98.48 | 105.45 | 111.33 | 128.40 | 152.67 | 109.61 | 89.73 |
| 19 | Benzyl alcohol | 761.04 | 856.50 | 855.81 | 834.49 | 837.47 | 721.46 | 798.17 | 832.46 | 755.95 | 789.60 | 675.34 | 698.37 | 713.49 | 723.50 | 816.65 | 930.86 | 1060.20 | 851.25 |
| 20 | 2,3-Cyclopentenopyridine | 10.53 | 11.19 | 9.68 | 11.45 | 9.21 | 10.39 | 9.61 | 11.34 | 6.82 | 7.87 | 5.91 | 7.24 | 5.30 | 6.35 | 5.53 | 7.50 | 5.82 | 4.05 |
| 21 | Benzeneacetaldehyde | 14.29 | 15.68 | 14.93 | 13.58 | 14.35 | 16.55 | 15.01 | 14.60 | 70.74 | 68.71 | 59.54 | 72.49 | 71.89 | 78.59 | 82.45 | 96.34 | 119.99 | 95.16 |
| 22 | Pantolactone | 11.48 | 13.89 | 11.59 | 11.67 | 13.49 | 12.40 | 12.28 | 11.39 | 9.72 | 10.62 | 7.23 | 9.48 | 8.87 | 7.40 | 8.86 | 10.61 | 7.50 | 6.97 |
| 23 | 3,5-Octadien-2-one | 3.06 | 2.77 | 2.73 | 1.75 | 2.81 | 2.35 | 3.61 | 2.84 | 4.13 | 3.76 | 3.06 | 6.35 | 3.58 | 5.35 | 4.20 | 3.42 | 4.76 | 3.55 |
| 24 | Acetophenone | 6.29 | 5.84 | 5.92 | 5.58 | 7.23 | 6.49 | 6.63 | 5.93 | 4.23 | 4.49 | 3.94 | 5.24 | 7.83 | 6.19 | 4.01 | 4.72 | 4.52 | 3.57 |
| 25 | 4-Methylbenzaldehyde | 2.32 | 2.36 | 2.62 | 2.43 | 2.66 | 2.56 | 2.26 | 2.19 | 1.11 | 1.86 | 1.67 | 1.99 | 2.11 | 1.39 | 1.80 | 2.32 | 2.02 | 1.80 |
| 26 | 4-Methylphenol | 55.40 | 64.38 | 74.72 | 70.23 | 65.58 | 66.36 | 69.39 | 72.39 | 32.82 | 41.92 | 30.86 | 35.69 | 37.38 | 39.35 | 40.21 | 47.43 | 32.10 | 24.82 |
| 27 | 3-Methylbenzaldehyde | 5.93 | 6.30 | 6.96 | 6.40 | 5.55 | 6.59 | 7.97 | 7.23 | 2.40 | 2.51 | 2.21 | 3.49 | 4.66 | 2.35 | 1.79 | 2.24 | 2.07 | 1.54 |
| 28 | 2-Methoxy phenol | 128.93 | 149.66 | 153.34 | 139.46 | 146.48 | 136.49 | 142.43 | 144.56 | 57.89 | 69.17 | 54.51 | 52.35 | 50.97 | 46.39 | 48.75 | 66.84 | 50.07 | 43.42 |
| 29 | Methyl benzoate | 7.07 | 3.59 | 7.24 | 6.83 | 4.74 | 5.39 | 5.40 | 6.36 | 4.94 | 5.00 | 4.90 | 5.49 | 4.06 | 4.59 | 3.45 | 4.32 | 5.02 | 3.66 |
| 30 | Ethyl 2-methylpropanoate | 9.62 | 14.39 | 11.11 | 11.70 | 11.47 | 10.38 | 10.72 | 12.38 | 6.26 | 7.28 | 4.84 | 5.49 | 3.99 | 4.23 | 5.68 | 6.89 | 3.51 | 2.51 |
| BlobID | Compound Name | 0h | 0h | 0h | 0h | 0h | 0h | 48h | 48h | 48h | 48h | 48h | 48h | 72h | 72h | 72h | 72h | 72h | 72h |
| 31 | 3-Acetylpyridine | 2.39 | 2.15 | 2.96 | 2.23 | 2.61 | 2.35 | 2.78 | 3.44 | 1.39 | 1.40 | 1.37 | 1.79 | 1.81 | 1.44 | 1.74 | 1.83 | 1.80 | 1.70 |
| 32 | 6-Methyl-3,5-heptadien-2-one | 5.06 | 7.35 | 5.25 | 4.57 | 6.24 | 6.44 | 5.78 | 6.35 | 2.18 | 4.83 | 4.09 | 5.08 | 4.10 | 5.15 | 4.42 | 4.54 | 4.75 | 3.46 |
| 33 | Nonanal | 22.06 | 23.46 | 20.07 | 20.40 | 21.94 | 24.66 | 20.89 | 20.43 | 40.17 | 26.22 | 23.71 | 25.48 | 26.83 | 28.49 | 28.91 | 31.79 | 25.51 | 26.03 |
| 34 | Linalool | 9.77 | 12.44 | 9.63 | 10.12 | 9.34 | 11.38 | 10.82 | 11.37 | 8.82 | 13.66 | 9.19 | 8.49 | 10.30 | 11.15 | 9.84 | 11.96 | 9.54 | 6.86 |
| 35 | Phenylethyl Alcohol | 1099.70 | 1299.55 | 1294.81 | 1106.02 | 1230.32 | 114.46 | 1200.17 | 124.50 | 818.86 | 861.20 | 713.53 | 800.39 | 714.28 | 785.60 | 816.23 | 949.40 | 838.06 | 691.61 |
| 36 | Methyl octanoate | 10.97 | 11.87 | 9.43 | 10.76 | 10.65 | 9.87 | 10.25 | 11.24 | 15.08 | 14.27 | 14.97 | 13.48 | 7.59 | 11.34 | 7.18 | 8.04 | 19.53 | 20.68 |
| 37 | Isophorone | 101.34 | 128.38 | 103.35 | 105.23 | 110.34 | 118.51 | 102.57 | 118.49 | 77.81 | 111.78 | 80.65 | 70.49 | 86.25 | 98.01 | 92.35 | 81.48 | 77.95 | 54.62 |
| 38 | 2-Dimethoxy- benzene | 14.70 | 16.79 | 15.78 | 18.31 | 10.37 | 16.24 | 17.16 | 15.39 | 10.10 | 11.62 | 9.20 | 11.33 | 6.26 | 9.48 | 7.37 | 6.27 | 9.27 | 5.75 |
| 39 | 4-Methyl-benzenemethanol | 4.85 | 4.09 | 4.48 | 3.76 | 4.11 | 4.69 | 5.13 | 3.86 | 3.01 | 2.09 | 2.63 | 2.88 | 2.92 | 2.66 | 3.13 | 3.75 | 3.51 | 2.36 |
| 40 | (E)-2-Nonenal | 1.58 | 1.63 | 1.62 | 1.50 | 1.65 | 1.66 | 1.78 | 1.71 | 3.36 | 2.01 | 1.75 | 3.24 | 2.17 | 2.38 | 2.20 | 2.47 | 2.31 | 2.02 |
| 41 | 1-Nonanol | 18.11 | 20.70 | 19.36 | 18.83 | 20.05 | 21.40 | 20.12 | 17.49 | 10.68 | 12.40 | 9.73 | 11.24 | 9.32 | 10.24 | 11.04 | 11.02 | 9.82 | 7.93 |
| 42 | 4-Ethylphenol | 280.90 | 303.31 | 355.52 | 282.16 | 324.24 | 176.50 | 342.65 | 298.45 | 208.38 | 218.63 | 176.20 | 234.86 | 207.13 | 194.58 | 242.24 | 272.94 | 196.39 | 142.14 |
| 43 | Decanal | 35.61 | 38.18 | 38.47 | 37.42 | 38.18 | 36.39 | 36.72 | 42.25 | 52.94 | 45.56 | 40.92 | 37.59 | 42.20 | 45.39 | 50.39 | 46.58 | 47.07 | 47.80 |
| 44 | Terpineol | 7.52 | 6.57 | 6.64 | 6.62 | 6.46 | 5.90 | 6.75 | 7.39 | 4.42 | 6.04 | 5.95 | 5.35 | 5.50 | 7.70 | 8.71 | 8.68 | 8.34 | 6.25 |
| 45 | Methyl 2-methylvalerate | 30.89 | 44.63 | 45.30 | 46.45 | 38.41 | 38.49 | 36.46 | 42.36 | 61.67 | 74.75 | 79.13 | 67.49 | 79.64 | 56.49 | 42.18 | 122.89 | 43.15 | 54.48 |
| 46 | 2-Phenoxyethanol | 25.68 | 32.65 | 81.02 | 44.26 | 45.29 | 65.29 | 48.52 | 56.29 | 38.71 | 35.38 | 33.49 | 42.49 | 49.60 | 36.40 | 40.27 | 78.70 | 38.12 | 43.63 |
| 47 | 2,5-Dimethylbenzaldehyde | 9.61 | 10.09 | 11.30 | 7.03 | 11.84 | 9.24 | 12.61 | 11.29 | 4.72 | 5.16 | 4.22 | 3.70 | 4.44 | 4.11 | 4.06 | 5.57 | 4.59 | 3.06 |
| 48 | 3-Ethyl-4-methylpyrrole-2,5-dione | 10.45 | 9.20 | 13.24 | 7.13 | 10.01 | 8.95 | 13.12 | 12.34 | 14.47 | 9.63 | 8.56 | 7.59 | 8.01 | 6.50 | 8.03 | 10.89 | 10.50 | 6.84 |
| 49 | 2,3-Dihydrobenzofuran | 33.37 | 31.22 | 46.07 | 34.72 | 37.47 | 36.24 | 38.17 | 40.38 | 24.65 | 25.51 | 22.61 | 31.39 | 31.02 | 28.50 | 35.24 | 38.91 | 25.34 | 14.78 |
| 50 | 2-Ethylhexyl acrylate | 118.58 | 117.69 | 111.01 | 118.86 | 115.21 | 117.38 | 113.61 | 121.23 | 65.62 | 67.85 | 69.93 | 56.39 | 84.27 | 84.35 | 87.06 | 134.70 | 64.70 | 79.52 |
| 51 | β-Cyclocitral | 5.07 | 6.56 | 5.65 | 5.23 | 6.13 | 5.92 | 5.87 | 5.69 | 5.62 | 7.23 | 5.73 | 5.40 | 5.91 | 6.37 | 5.92 | 6.54 | 6.23 | 4.86 |
| 52 | Benzothiazole | 19.06 | 18.03 | 33.12 | 17.46 | 23.76 | 21.38 | 29.65 | 25.35 | 19.69 | 21.45 | 18.66 | 17.38 | 23.26 | 21.38 | 19.78 | 27.35 | 20.36 | 21.71 |
| 53 | 2-Ethylhexyl propionate | 26.73 | 19.89 | 17.15 | 16.63 | 26.79 | 22.34 | 22.78 | 21.28 | 16.35 | 17.33 | 17.39 | 16.49 | 15.92 | 18.24 | 21.79 | 27.32 | 15.42 | 16.51 |
| 54 | 1-Decanol | 4.53 | 5.59 | 6.25 | 4.24 | 6.64 | 5.49 | 6.73 | 5.49 | 1.89 | 2.35 | 1.69 | 1.75 | 1.86 | 2.19 | 2.27 | 2.09 | 2.11 | 1.54 |
| 55 | Nonanoic Acid | 3.65 | 3.45 | 4.35 | 3.52 | 3.59 | 3.69 | 3.64 | 4.24 | 2.68 | 1.28 | 2.34 | 1.78 | 4.26 | 2.34 | 2.90 | 4.08 | 2.66 | 2.37 |
| 56 | 4-Ethyl-2-methoxyphenol | 94.39 | 102.52 | 119.84 | 121.64 | 104.32 | 104.50 | 95.04 | 98.35 | 50.21 | 61.84 | 47.31 | 56.35 | 48.78 | 52.33 | 44.06 | 57.08 | 54.01 | 34.24 |
| 57 | 3-Undecanone | 11.41 | 11.21 | 11.12 | 11.24 | 10.55 | 9.84 | 12.87 | 10.38 | 4.04 | 7.97 | 6.76 | 4.56 | 9.91 | 6.49 | 9.70 | 12.76 | 7.78 | 6.18 |
| 58 | Indole | 72.94 | 76.56 | 99.45 | 77.97 | 84.35 | 95.24 | 85.01 | 89.50 | 38.28 | 44.10 | 36.99 | 42.39 | 41.49 | 46.49 | 42.03 | 48.11 | 36.92 | 25.51 |
| 59 | Methyl Decanoate | 31.80 | 25.65 | 27.50 | 25.26 | 27.49 | 29.49 | 30.28 | 32.50 | 37.56 | 35.69 | 33.23 | 26.49 | 22.06 | 32.78 | 16.85 | 15.33 | 38.01 | 19.44 |
| 60 | 2-Methoxy-4-vinylphenol | 55.69 | 52.19 | 67.20 | 56.82 | 58.98 | 57.50 | 59.28 | 62.35 | 22.85 | 30.45 | 24.71 | 26.48 | 24.18 | 27.49 | 19.82 | 28.73 | 24.09 | 15.48 |
| 61 | Solanone | 361.13 | 389.99 | 419.24 | 388.83 | 366.81 | 385.40 | 413.61 | 403.49 | 341.95 | 386.33 | 332.49 | 312.24 | 258.70 | 264.60 | 249.19 | 214.21 | 312.16 | 238.07 |
| BlobID | Compound Name | 0h | 0h | 0h | 0h | 0h | 0h | 48h | 48h | 48h | 48h | 48h | 48h | 72h | 72h | 72h | 72h | 72h | 72h |
| 62 | Nicotine | 6611.05 | 6470.41 | 6947.19 | 6772.31 | 6583.76 | 6223.94 | 6679.65 | 6345.96 | 6410.35 | 6490.69 | 6414.57 | 6321.39 | 5816.28 | 6432.89 | 6530.12 | 7331.52 | 7940.77 | 6240.92 |
| 63 | 3-Methyl-1H-indole | 35.86 | 35.33 | 46.41 | 48.31 | 36.79 | 33.50 | 32.78 | 37.49 | 22.19 | 25.06 | 21.11 | 21.85 | 25.42 | 23.87 | 26.40 | 29.38 | 23.99 | 18.38 |
| 64 | Damascenone | 4.22 | 4.40 | 4.62 | 3.56 | 4.16 | 5.33 | 3.96 | 5.13 | 3.27 | 3.92 | 3.01 | 3.70 | 4.30 | 4.25 | 3.98 | 3.86 | 4.56 | 3.67 |
| 65 | β-Damascone | 11.82 | 11.94 | 13.61 | 11.67 | 12.54 | 13.36 | 11.50 | 13.25 | 3.75 | 11.11 | 8.88 | 7.98 | 9.23 | 9.05 | 8.28 | 8.52 | 10.54 | 7.58 |
| 66 | Myosmine | 170.69 | 166.57 | 221.21 | 183.20 | 188.64 | 178.43 | 186.73 | 185.68 | 129.34 | 125.27 | 148.28 | 132.90 | 126.25 | 142.39 | 148.24 | 159.55 | 143.98 | 172.92 |
| 67 | Geranyl acetone | 78.32 | 72.76 | 92.31 | 80.23 | 78.24 | 84.52 | 79.39 | 83.26 | 56.96 | 57.87 | 54.17 | 55.87 | 61.90 | 61.50 | 57.75 | 62.34 | 70.82 | 56.98 |
| 68 | Norsolanadione | 84.83 | 84.12 | 108.89 | 84.12 | 99.73 | 94.59 | 93.05 | 104.38 | 43.34 | 43.48 | 38.48 | 32.49 | 34.69 | 38.45 | 36.30 | 39.78 | 41.51 | 39.12 |
| 69 | Nicotyrine | 2304.46 | 2289.49 | 2587.26 | 2214.61 | 2503.08 | 2156.96 | 2340.56 | 2346.38 | 1709.37 | 1907.53 | 1884.03 | 1982.29 | 1673.70 | 1780.34 | 1905.45 | 2029.85 | 2109.52 | 1914.06 |
| 70 | α-Ionone | 18.74 | 17.99 | 21.41 | 17.65 | 18.95 | 20.64 | 17.89 | 21.77 | 14.47 | 16.84 | 13.61 | 12.85 | 15.17 | 14.40 | 13.88 | 15.24 | 20.81 | 13.74 |
| 71 | β-Ionol | 11.44 | 6.72 | 9.32 | 9.43 | 9.78 | 8.47 | 8.89 | 9.25 | 4.29 | 11.96 | 6.60 | 4.42 | 4.52 | 5.67 | 5.83 | 6.10 | 10.39 | 10.75 |
| 72 | 2,3'-Dipyridyl | 13.42 | 9.84 | 13.93 | 13.44 | 14.48 | 11.49 | 11.87 | 12.35 | 10.22 | 7.16 | 8.12 | 9.76 | 13.41 | 11.33 | 10.18 | 10.76 | 16.95 | 15.77 |
| 73 | Dihydroactinidiolide | 37.30 | 38.44 | 52.59 | 40.15 | 41.54 | 44.64 | 42.75 | 44.86 | 29.27 | 32.42 | 28.45 | 30.37 | 36.47 | 36.54 | 32.65 | 34.56 | 40.56 | 37.89 |
| 74 | Megastigmatrienone A | 0.27 | 0.24 | 0.32 | 0.23 | 0.25 | 0.35 | 0.26 | 0.31 | 0.11 | 0.11 | 0.17 | 0.08 | 0.12 | 0.09 | 0.07 | 0.05 | 0.13 | 0.06 |
| 75 | Megastigmatrienone B | 0.79 | 0.53 | 0.78 | 0.65 | 0.56 | 0.89 | 0.68 | 0.72 | 0.45 | 0.69 | 0.59 | 0.16 | 0.18 | 0.33 | 0.24 | 0.35 | 0.30 | 0.32 |
| 76 | Megastigmatrienone C | 0.21 | 0.19 | 0.21 | 0.17 | 0.21 | 0.23 | 0.19 | 0.21 | 0.13 | 0.21 | 0.18 | 0.16 | 0.26 | 0.29 | 0.19 | 0.22 | 0.23 | 0.20 |
| 77 | Megastigmatrienone D | 0.24 | 0.28 | 0.33 | 0.25 | 0.28 | 0.32 | 0.28 | 0.33 | 0.15 | 0.27 | 0.26 | 0.28 | 0.28 | 0.31 | 0.28 | 0.29 | 0.38 | 0.22 |
| 78 | 3-Hydroxy-β-damascone | 1.12 | 1.05 | 1.21 | 1.11 | 1.21 | 1.07 | 1.23 | 1.04 | 1.19 | 1.08 | 1.21 | 1.27 | 0.83 | 1.32 | 1.22 | 1.25 | 1.60 | 1.38 |
| 79 | 3-Oxo-a-ionol | 3.68 | 2.82 | 4.76 | 3.25 | 3.67 | 4.34 | 3.53 | 3.91 | 4.20 | 3.59 | 3.49 | 9.15 | 1.34 | 8.26 | 6.23 | 5.68 | 7.18 | 3.95 |
| 80 | Solavetivone | 9.34 | 8.58 | 11.69 | 9.23 | 10.24 | 10.24 | 9.46 | 10.21 | 2.85 | 2.94 | 2.50 | 2.27 | 2.32 | 2.34 | 2.52 | 2.22 | 2.68 | 1.70 |
| 81 | Neophytadiene | 1921.91 | 1911.54 | 1932.27 | 1866.24 | 1922.05 | 2158.38 | 1898.56 | 1948.36 | 1573.61 | 1371.10 | 1271.62 | 1255.71 | 1326.39 | 1354.08 | 1345.67 | 1385.43 | 1736.55 | 1694.44 |
| 82 | Hexadecanoic acid, methyl ester | 63.55 | 58.24 | 98.05 | 81.23 | 72.18 | 89.95 | 66.72 | 78.35 | 142.52 | 110.48 | 115.24 | 98.24 | 86.65 | 102.34 | 65.15 | 79.24 | 171.08 | 169.47 |
|  |  |  |  |  |  |  |  |  |  |  |  |  |  |  |  |  |  |  |  |

**Table S2.** Plastid pigment metabolites in tobacco leaves during curing.

| **Metabolites** | | **Curing time (h)** | | |
| --- | --- | --- | --- | --- |
| **Category** | **Compounds (ng.g^-1^)** | **0** | **48** | **72** |
| Carotenoid | 6-Methyl-5-hepten-2-ol | 2.03±0.09^a^ | 1.71±0.11^b^ | 1.67±0.09^b^ |
|  | *β*-Ionol | 9.16±0.47^a^ | 6.17±0.88^b^ | 8.34±0.65^a^ |
|  | 3-Oxo-a-ionol | 3.75±0.21^b^ | 5.24±0.92^ab^ | 5.96±0.72^a^ |
|  | Linalool | 10.61±0.38^a^ | 10.42±0.62^a^ | 9.30±0.48^a^ |
|  | 6-Methyl-5-hepten-2-one | 9.26±0.19^a^ | 4.69±0.16^b^ | 4.92±0.11^b^ |
|  | 6-Methyl-3,5-heptadien-2-one | 5.88±0.32^a^ | 4.30±0.33^b^ | 5.02±0.29^ab^ |
|  | Isophorone | 111.03±3.46^a^ | 87.35±4.61^b^ | 82.13±5.86^b^ |
|  | *β*-Damascenone | 4.42±0.21^a^ | 3.79±0.16^b^ | 4.84±0.21^a^ |
|  | *β*-Damascone | 12.46±0.30^a^ | 8.35±0.74^b^ | 9.38±0.33^b^ |
|  | Geranylacetone | 81.13±2.03^a^ | 58.54±1.07^b^ | 62.46±2.05^b^ |
|  | α-Ionone | 19.38±0.58^a^ | 14.56±0.43^c^ | 16.76±0.83^b^ |
|  | Megastigmatrienone A | 0.28±0.01^a^ | 0.10±0.01^b^ | 0.09±0.01^b^ |
|  | Megastigmatrienone B | 0.70±0.04^a^ | 0.37±0.07^b^ | 0.28±0.01^b^ |
|  | Megastigmatrienone C | 0.20±0.01^a^ | 0.21±0.02^a^ | 0.19±0.01^a^ |
|  | Megastigmatrienone D | 0.29±0.01^b^ | 0.27±0.02^b^ | 0.39±0.07^a^ |
|  | 3-Hydroxy-β-damascone | 1.13±0.03^b^ | 1.17±0.06^b^ | 1.54±0.05^a^ |
|  | Solavetivone | 9.87±0.33^a^ | 2.50±0.10^b^ | 2.10±0.10^b^ |
|  | *β*-Cyclocitral | 5.76±0.17^a^ | 6.09±0.21^a^ | 5.55±0.17^a^ |
|  | Dihydroactinidiolide | 42.78±1.70^a^ | 32.59±1.10^c^ | 37.60±1.06^b^ |
|  | Sum | 330.13±5.74^a^ | 248.43±8.29^b^ | 258.52±8.04^b^ |
| Chlorophyll | Neophytadiene | 1944.91±31.69^a^ | 1360.45±34.45^c^ | 1650.59±38.15^b^ |

Relative concentrations were analyzed using phenylethyl acetate as the internal standard. Data are shown as the means ± SEs (n = 8). Different letters indicate a significant difference (*P* < 0.05) between values during the yellowing stages (0h, 48h and 72h) based on Duncan’s multiple range test in SPSS.

**Table S3.** An overview of protein identification in tobacco leaves during curing.

| Sample name | Total spectra | Spectra | Unique Spectra | Peptide | Unique Peptide | Protein |
| --- | --- | --- | --- | --- | --- | --- |
| Run1 | 342013 | 121592 | 73673 | 24453 | 16000 | 3696 |
| Run2 | 348406 | 125150 | 73922 | 24956 | 16908 | 5140 |
| Run3 | 353259 | 125832 | 70786 | 24935 | 16323 | 3769 |
| Total | 1043678 | 372574 | 218381 | 30498 | 22993 | 5931 |

**Table S4.** DEPs involved in pigment metabolism and color change in tobacco leaves during curing.

| **Accession^a^** | **Protein description^b^** | | **Species^c^** | **Identity^d^** | **Mean ratio^e^** | | |
| --- | --- | --- | --- | --- | --- | --- | --- |
|  |  |  |  |  | **48 h vs 0 h** | **72 h vs 0 h** | **72 h vs 48 h** |
| Carotenoid biosynthesis | | | | | | | |
| gi\|697171377 | | Geranylgeranyl pyrophosphate synthase, chloroplastic-like | NTO | 100 | 0.29 | 0.24 | 0.90 |
| gi\|697095176 | | Zeaxanthin epoxidase, chloroplastic-like | NTO | 100 | 1.58 | 1.89 | 1.19 |
| gi\|698545986 | | Violaxanthin de-epoxidase, chloroplastic | NSY | 100 | 0.44 | 0.36 | 0.87 |
| gi\|257682854 | | Unnamed protein product | NTA | 100 | 0.71 | 0.53 | 0.73 |
| gi\|698430265 | | Scopoletin glucosyltransferase-like | NSY | 100 | 1.65 | 1.41 | 0.91 |
| Carotenoid cleavage | | | | | | | |
| gi\|698442977 | | Probable linoleate 9S-lipoxygenase 5 isoform X1 | NSY | 100 | 1.67 | 1.63 | 1.02 |
| gi\|697143875 | | Probable linoleate 9S-lipoxygenase 5 isoform X2 | NTO | 100 | 1.65 | 1.62 | 0.98 |
| gi\|697099543 | | Peroxidase 12-like | NTO | 100 | 0.62 | 0.64 | 1.01 |
| gi\|697105076 | | Peroxidase P7-like | NTO | 100 | 1.22 | 1.52 | 1.25 |
| gi\|698517935 | | Cationic peroxidase 1-like | NSY | 100 | 1.86 | 1.74 | 1.03 |
| gi\|697136459 | | Cationic peroxidase 2-like | NTO | 100 | 0.88 | 0.59 | 0.64 |
| gi\|698520812 | | Suberization-associated anionic peroxidase-like | NSY | 100 | 1.31 | 1.52 | 1.17 |
| gi\|697111963 | | Peroxiredoxin Q, chloroplastic-like | NTO | 100 | 0.51 | 0.62 | 1.22 |
| gi\|698508811 | | 2-Cys peroxiredoxin BAS1, chloroplastic-like | NSY | 100 | 0.69 | 0.66 | 0.99 |
| gi\|698502291 | | L-ascorbate peroxidase 3, peroxisomal-like | NSY | 100 | 0.46 | 0.42 | 1.00 |
| gi\|698519135 | | L-ascorbate peroxidase 3, peroxisomal isoform X2 | NSY | 100 | 0.48 | 0.48 | 1.00 |
| gi\|257707682 | | unnamed protein product | NTA | 100 | 0.59 | 0.68 | 1.16 |
| gi\|698579037 | | Probable glutathione peroxidase 2 | NSY | 100 | 0.51 | 0.56 | 1.09 |
| gi\|20138338 | | Probable phospholipid hydroperoxide glutathione peroxidase | NTA | 100 | 0.63 | 0.63 | 1.02 |
| Chlorophyll biosynthesis | | | | | | | |
| gi\|669253446 | | Delta-aminolevulinic acid dehydratase | NTA | 100 | 0.81 | 0.60 | 0.77 |
| gi\|697112377 | | Porphobilinogen deaminase, chloroplastic-like | NTO | 100 | 0.66 | 0.57 | 0.86 |
| gi\|697096570 | | Protoporphyrinogen oxidase, chloroplastic | NTO | 100 | 0.68 | 0.74 | 1.11 |
| gi\|328727190 | | Ferrochelatase isoform I | NTA | 100 | 1.46 | 1.8 | 1.26 |
| gi\|697148787 | | Ferrochelatase-2, chloroplastic isoform X1 | NTO | 100 | 0.63 | 0.51 | 0.81 |
| gi\|697166771 | | Magnesium-chelatase subunit ChlI, chloroplastic isoform X1 | NTO | 100 | 0.35 | 0.32 | 0.97 |
| gi\|698565439 | | Magnesium protoporphyrin IX methyltransferase, chloroplastic | NSY | 100 | 0.44 | 0.47 | 1.11 |
| **Accession^a^** | | **Protein description^b^** | **Species^c^** | **Identity^d^** | **Mean ratio^e^** | | |
|  |  |  |  |  | **48 h vs 0 h** | **48 h vs 0 h** | **48 h vs 0 h** |
| gi\|698580985 | | Magnesium-protoporphyrin IX monomethyl ester [oxidative] cyclase, chloroplastic | NSY | 100 | 0.42 | 0.41 | 0.93 |
| gi\|697164457 | | Uncharacterized protein ycf39 isoform X2 | NTO | 100 | 0.45 | 0.30 | 0.66 |
| gi\|698518647 | | Protein TIC 62, chloroplastic isoform X3 | NSY | 100 | 0.53 | 0.51 | 0.98 |
| gi\|743401798 | | Geranylgeranyl reductase | NBE | 100 | 0.58 | 0.52 | 0.93 |
| Chlorophyll breakdown | | | | | | | |
| gi\|698580465 | | Chlorophyllase-1-like isoform X2 | NSY | 100 | 0.83 | 2.56 | 3.18 |

^a^Protein gi number from NCBI.

^b^Name of the protein identiﬁed by LC-MS/MS.

^c^Plant species from NCBI. NTO, NSY, NTA, NBE indicate *Nicotiana tomentosiformis*, *Nicotiana sylvestris*, and *Nicotiana tabacum*, *Nicotiana benthamiana*, respectively.

^d^Identity score of the BLAST (NCBInr).

^e^The ratio between protein levels at 48 h and 0 h, 72 h and 0 h or 72 h and 48 h in tobacco leaves.

**Table S5.** Primers used for qRT-PCR.

| **Gene name** | **Gene ID** | **Primer sequence** | **Product size (bp)** |
| --- | --- | --- | --- |
| *ZEP* | 104103833 | F 5'-GAACTGTGCTCGTGGGAGAT-3' | 212 |
|  |  | F 5'-TCCCACGCGATTTGCCATTA-3' |  |
| *VDE* | 104218706 | F 5'-AGGGTAGTGGGAGGCTGTAA-3' | 160 |
|  |  | F 5'-TGTTTTGTGGATGAGCTCCAA-3' |  |
| *LOX-5-X1* | 104215707 | F 5'-GGGGATCTTTCCACCATGTTCT-3' | 249 |
|  |  | F 5'-GGTTCTGCATGGTCAGCACT-3' |  |
| *POD-12* | 104084580 | F 5'-AGGCAAGGGCTTTTCACTTCT-3' | 151 |
|  |  | F 5'-CACCGGTCAACACGTTCATTT-3' |  |
| *PBGD* | 104103836 | F 5'-TCGAGGCGAAGATGGAGACT-3' | 222 |
|  |  | F 5'-GATCTCTCTGCCCGTAGTGT-3' |  |
| *CHLI* | 104089103 | F 5'-CTCGCTCCTCTAAATCTGCCG-3' | 178 |
|  |  | F 5'-GCAAGTTTCTGACCCTGTTCT-3' |  |
| *ChlM* | 104223503 | F 5'-GGACCTTCAAAGGCTACACGA-3' | 205 |
|  |  | F 5'-TCTAACCAGCGGCAAAGAAAC-3' |  |
| *Chlase-1-X2* | 104226899 | F 5'-TTCATTGGGTGAACACTCTGTA-3' | 173 |
|  |  | R 5'-GGGATGGCCCAGTTGGTTT-3' |  |
| *Actin* | 107800553 | F 5'-AGGGAAATTGTGAGGGACGT-3' | 168 |
|  |  | R 5'-AAGAACTTCTGGGCATCGGA-3' |  |

F: Forward primer; R: Reversed primer.


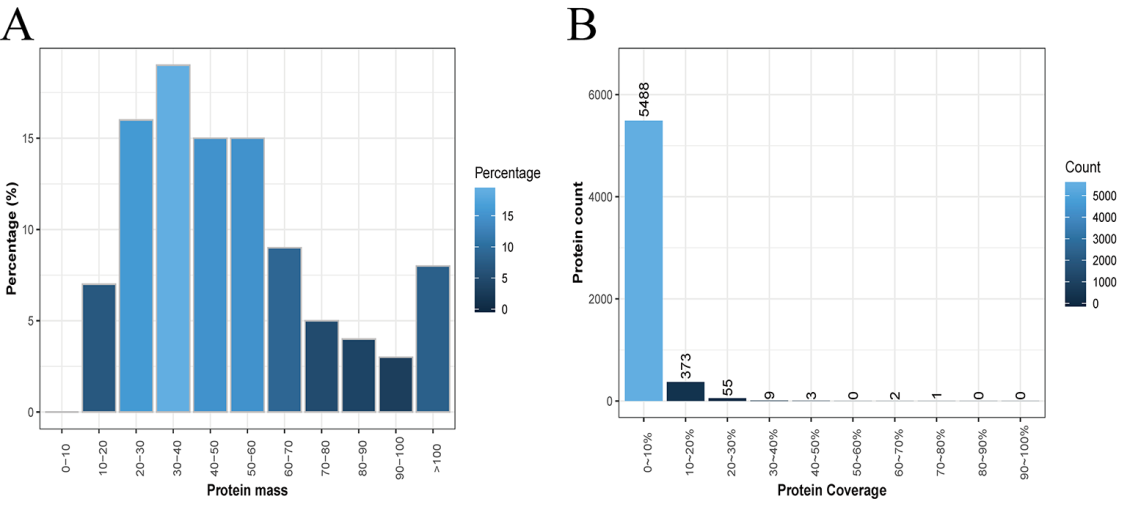


**Figure S1.** Protein mass distribution in tobacco leaves (**A**) and coverage of the proteins by the identified peptides (**B**).


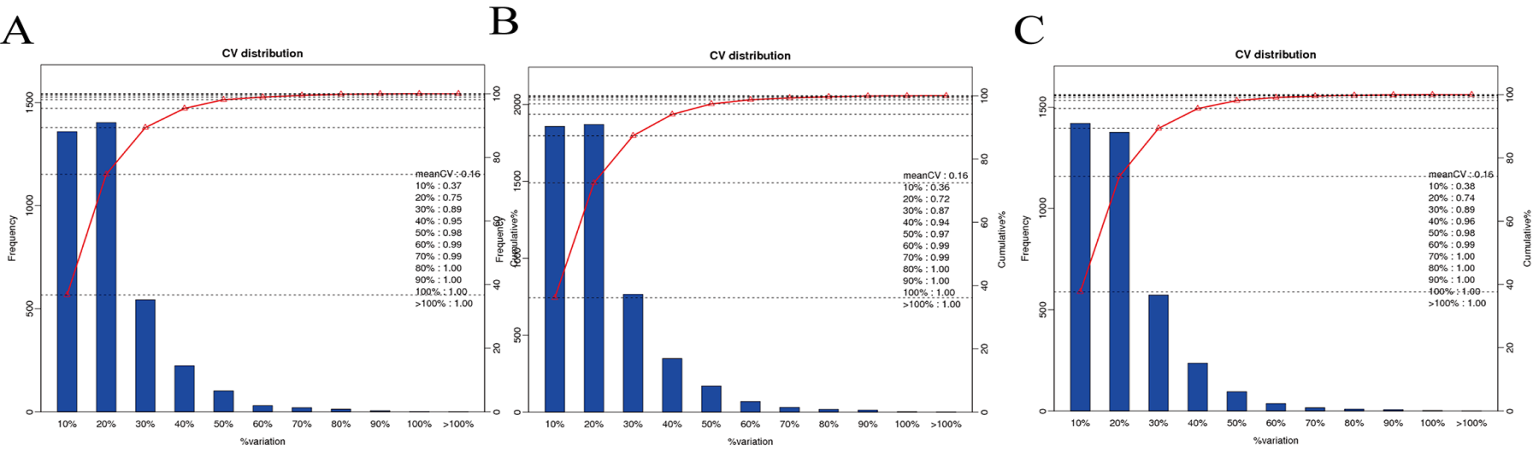


**Figure S2.** The distribution of coefficient of variation in three replicates. (**A**) Replicate 1 of different leaf samples; (**B**) replicate 2 of different leaf samples; (**C**) replicate 3 of different leaf samples. X-axis is the deviation between the protein ratio of the repetitive samples. Y-axis is the percentage that protein at a certain angle comprise quantified protein amount.


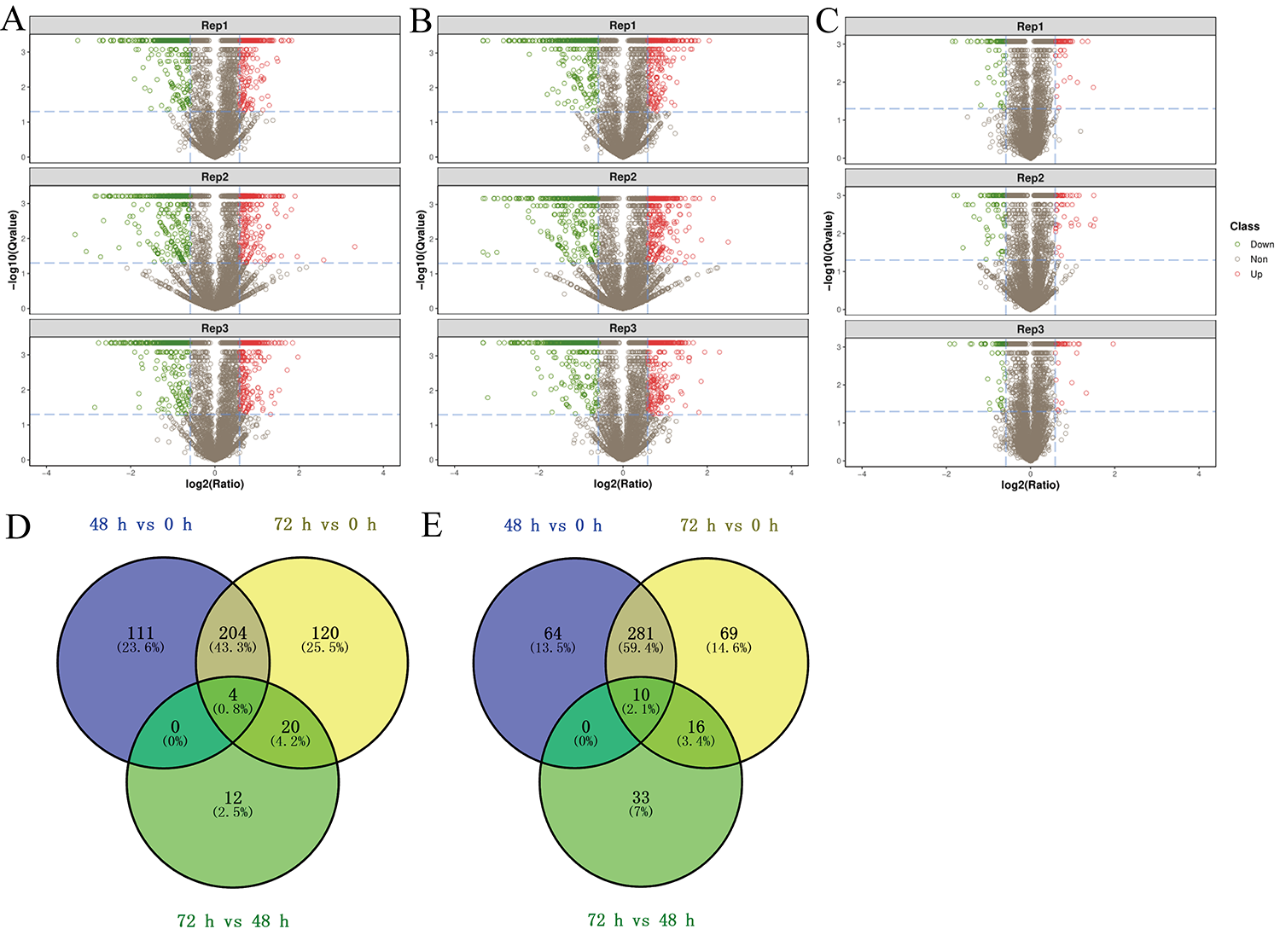


**Figure S3.** Volcano plots and Venn diagram of differentially expressed proteins in three replicates. (**A**) 48 h vs 0 h; (**B**) 72 h vs 0 h; (**C**) 72 h vs 48 h; (**D**) up-regulated proteins; (**E**) down-regulated proteins. This plot depicts volcano plot of log2 fold-change (x-axis) versus -log10 Qvalue (y-axis, representing the probability that the protein is differentially expressed). Qvalue <0.05 and Fold change > 1.5 are set as the significant threshold for differentially expression. The red and green dots indicate points-of-interest that display both large-magnitude fold-changes as well as high statistical significance. Dots in red mean significant up-regulated proteins which passed screening threshold. Dots in green mean significant down-regulated proteins which passed screening threshold. And gray dots are non-significant differentially expressed protein. The numbers of DEPs identiﬁed from three biological replicates are shown in the different segments.


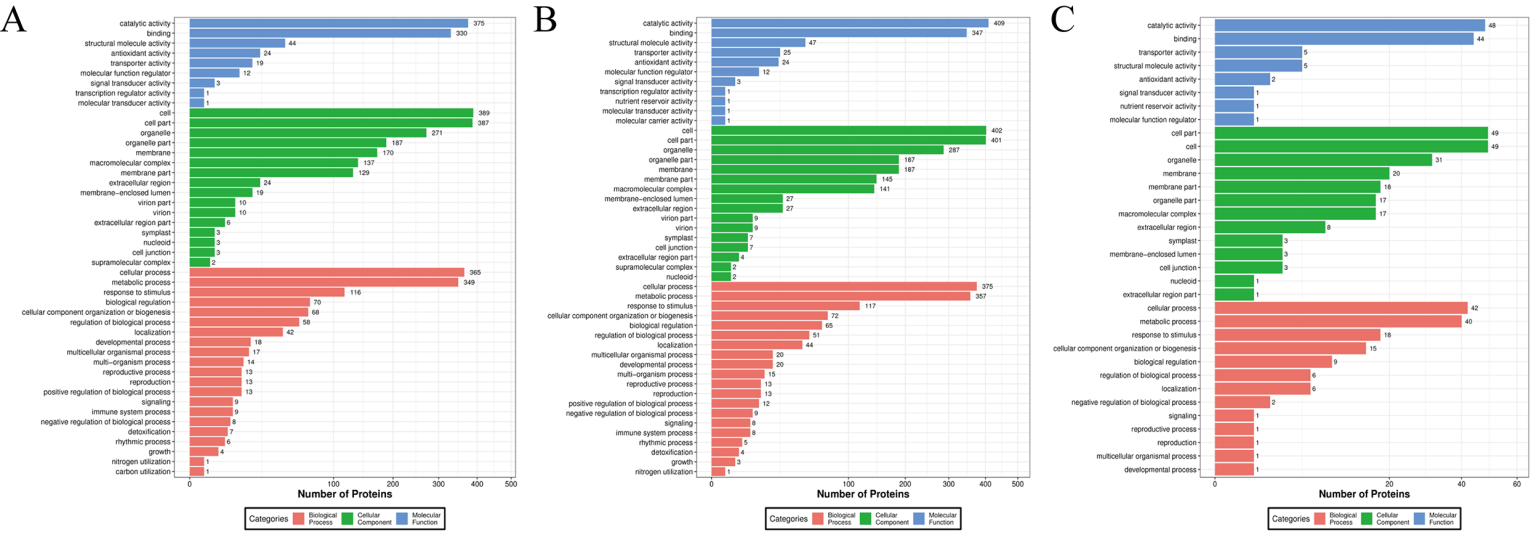


**Figure S4.** Barplot of the Gene Ontology analysis of DEPs obtained from different comparisons in tobacco leaf samples. (**A**) 48 h vs 0 h; (**B**) 72 h vs 0 h; (**C**) 72 h vs 48 h. X-axis displays protein count; y-axis displays GO term.


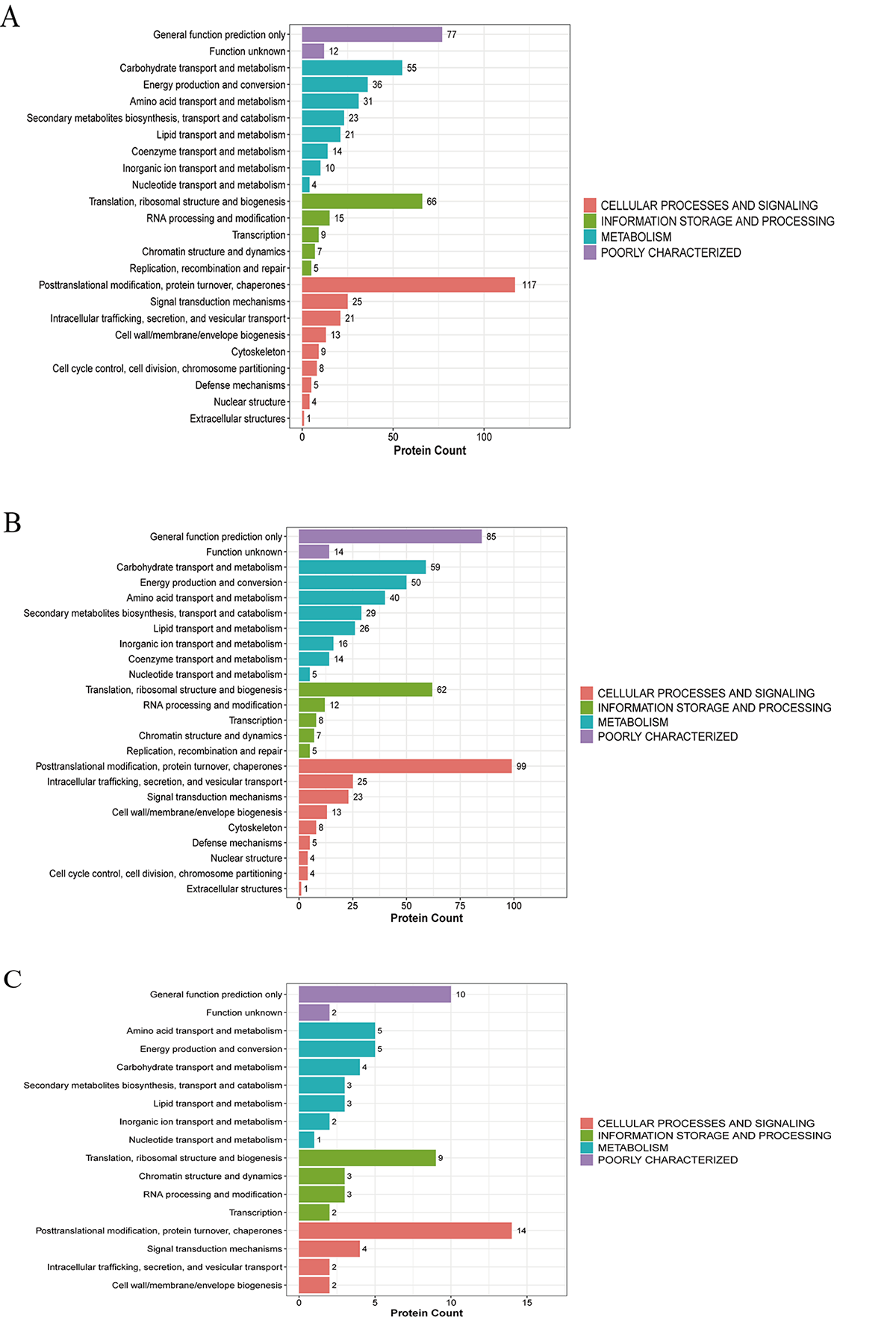


**Figure S5.** KOGs categories of DEPs obtained from different comparisons in tobacco leaf samples. (**A**) 48 h vs 0 h; (**B**) 72 h vs 0 h; (**C**) 72 h vs 48 h. Y-axis displays the KOG term; x-axis displays the corresponding protein count illustrating the protein number of different functions.


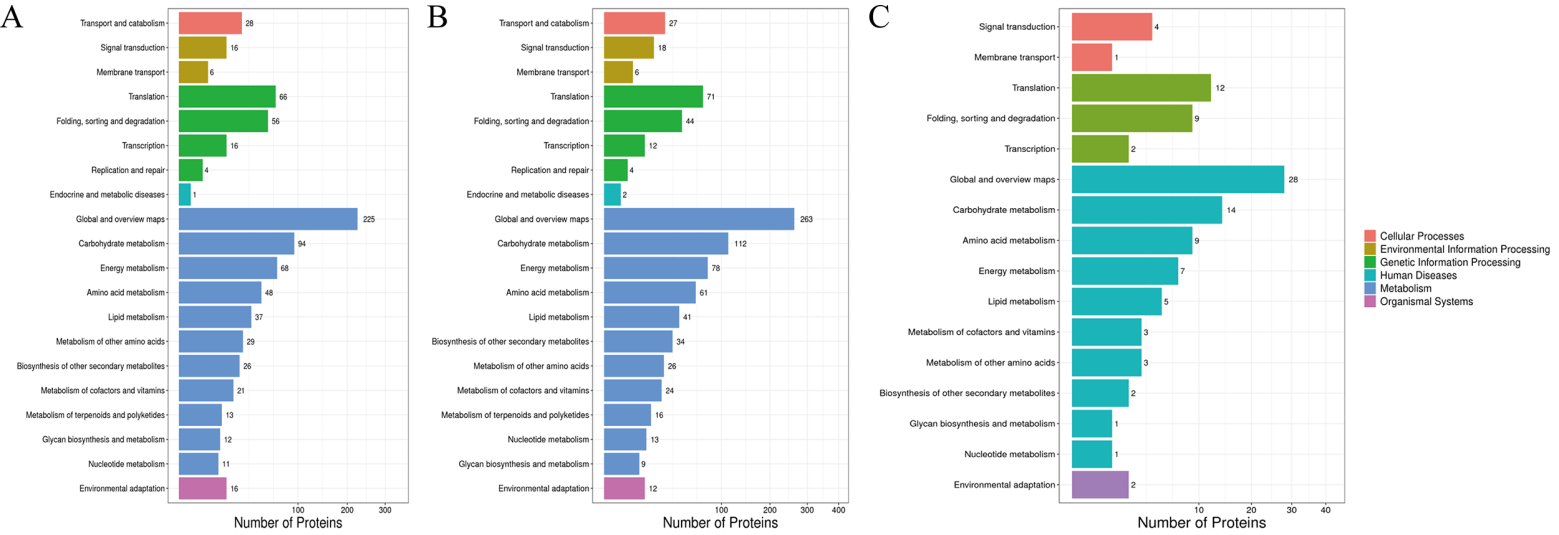


**Figure S6.** Barplots of KEGG pathway analysis of DEPs obtained from different comparisons in tobacco leaf samples. (**A**) 48 h vs 0 h; (**B**) 72 h vs 0 h; (**C**) 72 h vs 48 h. X-axis displays corresponding protein count; y-axis displays pathway name.

**Figure S7.** Curing schedule of tobacco leaves.

| - Temperature/°C   40  70  60  30  50 | Yellowing stage | | | | | | | Leaf-drying stage | | | | | Stem-drying stage | | | |
| --- | --- | --- | --- | --- | --- | --- | --- | --- | --- | --- | --- | --- | --- | --- | --- | --- |
|  |  |  |  |  |  |  |  | |  |  |  |  | |  | 68 °C |  |
|  |  |  |  |  |  |  |  | |  |  |  | 60 °C | | Dry-bulb temperature |  |  |
|  |  |  |  |  |  |  | 46–48 °C | |  | 52–54 °C |  |  | |  |  |  |
|  | 34–35 °C |  |  | 38 °C |  | 40–42 °C |  | |  |  |  |  | | Wet-bulb temperature |  |  |
|  | 33 °C |  | 35–36 °C |  | 33–34 °C |  | 35–36 °C | |  |  | 36–37 °C |  | | 38–39 °C |  |  |
|  |  |  |  |  |  |  |  | |  |  |  |  | |  |  |  |
| Curing time (h) 10 20 30 40 50 60 70 80 90 100 110 120 130 140 | | | | | | | | | | | | | | | | |
